# A cautionary note on the use of the Analysis of Covariance (ANCOVA) in classification designs with and without within-subject factors

**DOI:** 10.3389/fpsyg.2015.00474

**Published:** 2015-04-21

**Authors:** Bruce A. Schneider, Meital Avivi-Reich, Mindaugas Mozuraitis

**Affiliations:** Department of Psychology, University of Toronto MississaugaMississauga, ON, Canada

**Keywords:** ANCOVA, classification design, within-subject design, between-subjects design, mixed design

## Abstract

A number of statistical textbooks recommend using an analysis of covariance (ANCOVA) to control for the effects of extraneous factors that might influence the dependent measure of interest. However, it is not generally recognized that serious problems of interpretation can arise when the design contains comparisons of participants sampled from different populations (classification designs). Designs that include a comparison of younger and older adults, or a comparison of musicians and non-musicians are examples of classification designs. In such cases, estimates of differences among groups can be contaminated by differences in the covariate population means across groups. A second problem of interpretation will arise if the experimenter fails to center the covariate measures (subtracting the mean covariate score from each covariate score) whenever the design contains within-subject factors. Unless the covariate measures on the participants are centered, estimates of within-subject factors are distorted, and significant increases in Type I error rates, and/or losses in power can occur when evaluating the effects of within-subject factors. This paper: (1) alerts potential users of ANCOVA of the need to center the covariate measures when the design contains within-subject factors, and (2) indicates how they can avoid biases when one cannot assume that the expected value of the covariate measure is the same for all of the groups in a classification design.

## Introduction

It is commonplace in Psychology to compare the performance of participants randomly sampled from two or more mutually-exclusive groups. For instance, the ability of men to perform a particular task might be compared to that of that of women, or the ability of hearing-impaired individuals to remember details from a lecture they heard might be compared to that of individuals without hearing impairments. Such designs are often referred to as classification designs because participants are classified into two or more mutually-exclusive groups based on specific criteria (gender, hearing status, age, etc.). Once performance measures have been acquired on the participants from these different groups, the basis for their classification into different groups (e.g., gender, hearing status, age) is treated as a between-subjects factor in subsequent statistical analyses.

Psychologists also often favor within-subject designs (repeated measures designs) to explore the effects of fixed values of an independent variable on performance. For example, one could assess the speech recognition abilities of younger and older adults under different levels of noise. In such a design, Age would be a between-subjects classification factor, and Noise Level a within-subject experimental factor. The present paper identifies some pitfalls to be avoided when attempting to use an analysis of covariance (ANCOVA) in between-subjects classification designs, within-subject experimental designs, and in mixed designs in which one or more factors is classificatory, while other factors are within-subject.

### Between-subjects classification designs

In between-subjects classification designs, participants are randomly sampled from mutually-exclusive populations (e.g., men and women), giving rise to the different levels of a between-subjects classification factor (e.g., male vs. female). Such designs are to be contrasted with so-called experimental designs in which participants are randomly sampled from a population, and randomly assigned to different experimental conditions. For example, an experimenter might want to find out the extent to which the aggressive tendencies of adolescent males are modulated by the presence or absence of adolescent females. In such designs, half of the young men in the sample could have their aggressive tendencies assessed in the presence of young women whereas the other half have their aggressive tendencies assessed without young women being present. Here the presence or absence of young women when young men are being assessed for aggressive tendencies becomes an experimentally-defined, between-subjects factor in any subsequent statistical analyses.

In both experimental and classification designs it is understood that task performance might be affected by a number of different participant characteristics, such as their IQ, years of education, etc. It follows that if one could remove the contribution of individual differences on one or more of these characteristics to performance, one could more accurately assess the effects of the main factors of interest in the experiment. ANCOVA was specifically designed to do precisely this. Specifically, entering a covariate (such as IQ) into the analysis of an experimental design allows the experimenter to remove the contribution of the covariate to performance. This is the reason why a number of statistical textbooks recommend using an ANCOVA in experimental designs to control for the effects of extraneous factors that might influence the dependent measure of interest (e.g., Rutherford, 2011).

It is also widely-known and understood that an ANCOVA is based on the assumption that the relationship between the dependent variable and the covariate is linear, and that the slope of the line relating the dependent variable to the covariate does not differ across the different conditions in the experiment. For this reason, statistical textbooks recommend that the homogeneity of slope assumption be tested before conducting an ANCOVA. However, it is not so widely-known that the validity of an ANCOVA also depends on another assumption that is, by definition, valid for experimentally-defined between-subjects factors, but not necessarily for factors based on the classification of participants into mutually-exclusive groups. This assumption is that the expected value of the covariate is the same for all of the participants in the experiment. In experimental designs participants are randomly sampled from the same population and randomly assigned to the different levels of the between-subjects factor. Therefore, the expected value of a covariate measure taken on a participant will be the same for the different levels of the between-subjects factor. However, when a between-subjects factor is based on a classification of participants, this assumption does not necessarily hold. Hence, caution should be employed when considering an ANCOVA when one or more of the between-subjects factors are based on a classification of participants into different groups^1^.

To see why this is the case, consider the general linear model for a single factor, between-subjects design with one covariate measure taken on each of the participants, and only two levels of the between-subjects factor^2^. The general linear model of how the dependent variable (*y*) might be influenced by the two levels of the between-subjects factor and a single covariate (*x*) is:

(1) $$\begin{array}{l} y_{1,k}\text{​​​}=\mu +B+\alpha \left(x_{1,k}-\mu_{x1}\right)+e_{1,k}\\ y_{2,k}\text{​​​}=\mu -B+\alpha \left(x_{2,k}-\mu_{x2}\right)+e_{2,k} \end{array}$$

where the first subscript specifies the level of the between-subjects factor, and the second subscript the *k*th subject in that level (1 ≤ *k* ≤ *n*), with *n* subjects in each of the two groups. The grand mean in the population is μ, *B* is the additive effect associated with group 1 of the between-subjects factor, the *x*_1, *k*_ and *x*_2, *k*_ are normally-distributed covariate measures on the subjects in groups 1 and 2 of the between-subjects factor, α is the slope of the function relating the dependent variable *y*_*i, k*_ to the covariate, *e*_*i, k*_ is a normally-distributed error term whose mean is zero and whose standard deviation is σ_*wg*_, where σ_*wg*_ represents the joint contribution of both within-subject and between-subject error (σ^2^_*wg*_ = σ^2^_*ws*_ + σ^2^_*bs*_). Finally, for the sake of simplicity let us assume that the population standard deviations of the covariates in the two groups are equal (σ_*x*1_ = σ_*x*2_ = σ_*x*_), but allow the population means of the covariates in the two groups to differ one from another (μ_*x*1_ ≠ μ_*x*2_).

Note that there are two sources of variability in this model: within-group variance, and variance in the covariate measures. Hence, when α ≠ 0, the error term in a standard ANOVA (without the covariate) will reflect both sources of variance. The advantage of an ANCOVA is that it can remove the source of variance due to the covariate when evaluating between-subjects effects when certain conditions are met.

To determine the boundary conditions under which an ANCOVA can remove the source of variance due to the covariate, and legitimately test for mean level differences between the two groups (test the null hypothesis that *B* = 0), we need to determine the expected values of the various sums of squares for a standard ANCOVA of a two-level, between-subjects design^3^. In Table 1 we have done this for two cases: (1) μ_*x*1_ = μ_*x*2_; and (2) μ_*x*1_ ≠ μ_*x*2_. Table 1A presents the expected values of the sums of squares in this design when μ_*x*1_ = μ_*x*2_. As the Table 1A shows, an ANCOVA of data conforming to this model accomplishes three things. First, it removes any contribution arising from variability in the covariate from the error term used to test null hypotheses (the expected value of the mean square error term reflects only within-group variance, σ^2^_*wg*_). This increases the precision of the tests of statistical significance provided by the ANCOVA. Second, the expected value of the mean square for the between-subjects effect is a joint function of *n*, *B*, and σ^2^_*wg*_. Hence, the ratio of the mean square between-subjects to the mean square error provides a valid test of the null hypothesis that the mean difference between the two levels of the between-subjects factor is 0 (a valid test of H0: *B* = 0). Third, the ratio of the mean square for the covariate to the mean square error provides a valid test of whether the dependent variable and the covariate are correlated. Hence, an ANCOVA is clearly beneficial for evaluating between-subjects effects whenever the relationship between the covariate and the dependent variable does not vary across conditions, and μ_*d*_ = μ_*x*1_ − μ_*x*2_ = 0. Note that this assumption will always be valid when the subjects associated with the different between-subjects' levels are randomly selected from the same population and randomly assigned to different experimental conditions.

**Table 1:** **(A) Expected values of the Mean Squares for an ANCOVA analysis of a two-level, Between-Subjects Experiment for data characterized by Equation (1), when μ_*d*_ = μ_*x*1_- μ_*x*2_ = 0. Because covariate measures are automatically centered (mean covariate score subtracted from each covariate score) across all subjects when using one of the standard ANCOVA statistical packages, the experimenter does not need to center them when entering the data. (B) Expected Values of the Mean Squares for an ANCOVA for data characterized by Equation (1), when μ_*d*_ ≠ 0 (μ_*x*1_ ≠ μ_*x*2_). An ANCOVA should always be used to test the null hypothesis that α = 0 because the expected values of the Mean Square for the Covariate and the Mean Square for Error are the same independent of whether or not μ_*d*_ = 0. Note: *PDF_NCF_* is the non-central *F* distribution with *df*_1_ = 1, *df*_2_ = 2(*n* − 1), and non-centrality parameter, λ = (*n*μ^2^_*d*_)/(2σ^2^_*x*_), where *n* is the number of participants in each group. (C) Expected Values for an ANOVA of the data. An ANOVA should be used to test for the Main Effect when μ_*d*_ ≠ 0 (μ_*x*1_ ≠ μ_*x*2_)**.

**(A)**			
**Source**	***df***	**ANCOVA: E[Mean Square] when μ_*d*_ = 0**	***F***
Between	1	$\sigma_{wg}^{2}+\frac{4n\left(n-1\right)B^{2}}{2n-1}$	$\frac{MS_{Between}}{MS_{Error}}$ is a valid test of *H*0*: B* = 0
Covariate	1	σ^2^_*wg*_ + 2(*n* − 1)α^2^σ^2^_*x*_	$\frac{MS_{Covariate}}{MS_{Error}}$ is a valid test of *H*0: α = 0
Error	2n–3	σ^2^_*wg*_	
**(B)**			
**Source**	***df***	**ANCOVA: E[Mean Square] when μ_*d*_ ≠ 0**	***F***
Between	1	$\sigma_{wg}^{2}+\int_{x\;=\;0}^{\infty }\frac{n\left(n-1\right){\left(2B-\alpha \left(\mu_{x1}-\mu_{x2}\right)\right)}^{2}}{2\left(n-1\right)+f}PDF_{NCF}[df1,df2,\lambda ,f]df$	$\frac{MS_{Between}}{MS_{Error}}$ is not a valid test of *H*0: *B* = 0
Covariate	1	σ^2^_*wg*_ + 2(*n* − 1)α^2^σ^2^_*x*_	$\frac{MS_{Covariate}}{MS_{Error}}$ is a valid test of *H*0: α = 0
Error	2n–3	σ^2^_*wg*_	
**(C)**			
**Source**	***df***	**ANOVA: E[Mean Square]**	***F***
Between	1	σ^2^_*wg*_ + α^2^σ^2^_*x*_ + 2*nB*^2^	$\frac{MS_{Between}}{MS_{Error}}$ is a valid test of *H*0: *B* = 0
Error	2n–2	σ^2^_*wg*_ + α^2^σ^2^_*x*_	

The Table 1B shows how a violation of the assumption that μ_*d*_ = 0 affects tests of significance in an ANCOVA. Note that the expected value of the mean square error is the same independent of whether or not the covariate means (expected values of the covariate) in the two populations are equal, as is the mean square for the covariate. Hence, the ratio of the mean square for the covariate to the mean square error is a valid test of the hypothesis that α = 0, independent of any differences in the population mean values of the covariate in the two groups. However, the statistical test of the main effect provided by the ANCOVA when μ_*d*_ ≠ 0 (μ_*x*1_ ≠ μ_*x*2_) has a non-central F-Distribution with a centrality parameter that is a function of μ_*d*_ and σ_*x*_. Hence in classification designs, the statistical test for the between-subject main effect is not valid unless μ_*d*_ = 0 (μ_*x*1_ = μ_*x*2_). An examination of the expected value of the mean square for the between-subjects main effect when μ_*d*_ ≠ 0 indicates that the probability of a Type I error, when there is a correlation between the dependent variable and the covariate, will be higher than the nominal value chosen. The reason for this is that the covariate contributes to this main effect even when *B* = 0. Conversely, this same mean square indicates that a strong main effect (*B* > 0) will be reduced whenever α(μ_*x*1_ − μ_*x*2_) ≈ 2*B*, thereby reducing the power to detect a difference between the two groups when, in fact, there is one.

The Table 1C shows that a standard ANOVA provides a valid test of whether the expected difference between the two groups is significantly different from 0 even when μ_*d*_ ≠ 0 (μ_*x*1_ ≠ μ_*x*2_). This leads to the following recommendations when considering applying an ANCOVA. If participants are randomly assigned to the levels of a between-subjects factor, then conduct a standard ANCOVA. If, however, one or more of the factors is classificatory, use an ANCOVA to evaluate the overall contribution of any covariates. Then, use a standard ANOVA to evaluate other between-subjects effects.

To better understand the implications of performing an ANCOVA when there is, and when there is not, a difference between the expected values of the covariate in the two populations, consider the following concrete example. Suppose we are interested in how well native and non-native listeners can comprehend speech in different types of listening conditions. Because the literature has established a link between listening comprehension and reading comprehension, it makes sense to measure both, and use reading comprehension as a covariate when analyzing how acoustic variables affect listening comprehension. Research in our lab (Avivi-Reich et al., 2014) has shown that young adults whose first language is English (native speakers) have average Nelson-Denny reading comprehension scores of approximately 25, with a standard deviation of 6, whereas young adults for whom English is a second language (non-native speakers) have average Nelson-Denny reading comprehension scores of approximately 17, also with a standard deviation of 6. Because both listening and reading comprehension scores are likely to draw on a common pool of linguistic and cognitive processes, we would expect them to be correlated. In our lab we typically find the correlation coefficient between listening and reading comprehension scores to be about 0.4 in both populations when listening occurs in a quiet background. If we assume that these parameters characterize the distributions from which the participants were sampled, we can construct a model in which: (a) listening comprehension is correlated with reading comprehension scores to the same extent in both groups (ρ = 0.4); (b) the population standard deviation of the covariate measure (reading comprehension) is the same in both groups (σ_*x*_ = 6); (c) but the means of the covariates differ between the two group (μ_*x*1_ = 17, μ_*x*2_ = 25). Furthermore, we will make the assumption that pairs of scores (listening comprehension and reading comprehension) are bi-normally distributed.

**Figure 1:**
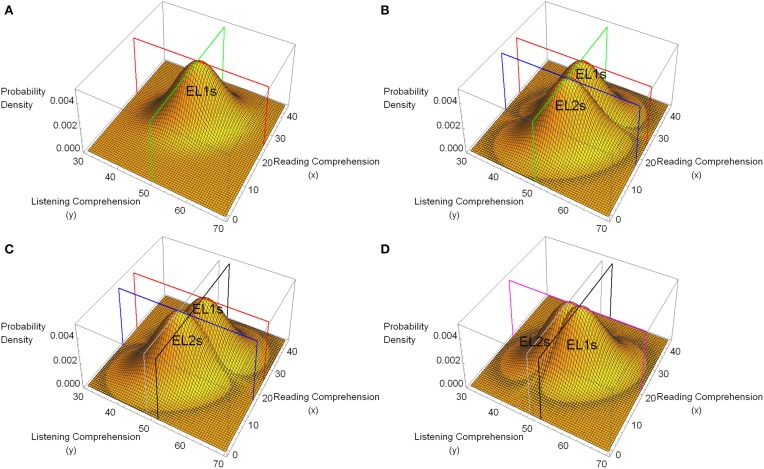
**Hypothetical bi-normal distributions of pairs of listening comprehension (dependent variable, *y*) and reading comprehension (covariate, *x*) scores for a population of native speakers for whom English is a first language (EL1s) and non-native speakers for whom English is a second language (EL2s)**. In all four plots the population correlation coefficient between listening and reading comprehension is 0.4 for both native speakers (EL1s) and non-native speakers (EL2s). **(A)** A hypothetical bi-normal distribution for native speakers (EL1s) with population mean value for listening comprehension of 50 (*SD* = 6), and reading comprehension of 25 (*SD* = 6). The green line defines the plane for *y* = 50, the red line for the plane *x* = 25. **(B)** The same distribution for the native speakers (EL1s) as in **(A)** along with the hypothetical distribution of data for non-native speakers (EL2s). Non-native speakers (EL2s) differ from native speakers (EL1s) only insofar as the population mean for their covariate measures is 17 instead of 25. The blue line outlines the plane for *x* = 17. **(C)** The mean of the *y* values for the native speakers (EL1s) here is 51.6, whereas it is 48.4 for the non-native speakers (EL2s). All of the other parameters are the same as in **(B)**. The gray line outlines the plane for *y* = 48.4, whereas the black line outlines the plane for *y* = 51.6. **(D)** The mean value of the covariate measure (reading comprehension) has been set to 21 for both groups. All other parameter values are the same as in **(C)**. The purple line outlines the plane corresponding to *x* = 21.

Figure 1A plots the hypothetical bi-normal distribution associated with the native speakers for the case in which the hypothetical population mean score for listening comprehension is 50, the population mean score for reading comprehension is 25, with the same standard deviation (6) for both measures, and a correlation between them of 0.4. As this distribution shows, higher listening comprehension scores tend to be associated with higher reading comprehension scores, and vice versa. Figure 1B plots this distribution along with a hypothetical distribution of paired listening and reading comprehension scores for the non-native speakers. In the non-native speakers' distribution, the mean value of the listening comprehension scores is assumed to be the same as for the native speakers (μ_*L, native speakers*_ = μ_*L, non−native speakers*_ = 50). However, the mean scores for reading comprehension are assumed to differ between the two groups (μ_*R, native speakers*_ = 25, μ_*R, non−native speakers*_ = 17), with the standard deviation of the scores along each dimension being the same in both groups for both reading and listening comprehension (σ_*L, native speakers*_ = σ _*L, non−native speakers*_ = σ _*R, native speakers*_ = σ _*R, non−native speakers*_ = 6). Finally, the correlation between listening and reading comprehension is assumed to be the same in both groups (ρ = 0.4).

Equation (1) specifies the general linear model that is equivalent to the bi-normal models depicted in Figure 1. Note that in the general linear model, the parameter α specifies the degree to which the covariate (in our example, the covariate is reading comprehension) contributes to the dependent variable (listening comprehension), whereas μ + *B* corresponds to the expected value of the dependent variable for group 1 (non-native speakers), and μ − *B* corresponds to the expected value for group 2 (native speakers)^4^. Hence, the two null hypotheses that we would like to test are *B* = 0, and α = 0.

In Figure 1B the green line outlines the plane defined by *y* = 50. Clearly, in this example, the mean listening comprehension score in both native speakers and non-native speakers is 50. Hence in this example *B* = 0. The blue line outlines the plane defined by *x* = 17, whereas the red lines outline the plane defined by *x* = 25. These planes clearly indicate that the mean covariate value for the non-native speakers' group is 17, whereas it is 25 for the native speakers' group. To simulate a two group experiment based on the model shown in Figure 1B, we took random samples of size 40 from each distribution, labeled the listening comprehension score as the dependent variable, and the reading comprehension score as the covariate, and conducted both an ANCOVA and ANOVA analysis of the data. Assuming a Type I error of 0.05, we determined whether or not the null hypothesis that there was no main effect due to Group (*B* = 0, no differences between the native and non-native speakers) was rejected for the ANCOVA and ANOVA. We repeated this procedure 10,000 times and counted the number of rejections of this null hypothesis for both types of analyses. The null hypothesis that there was no main effect due to group (no differences between the native and non-native speakers) was rejected approximately 5% of the time when an ANOVA was conducted on the data (consistent with a Type I error of 0.05), but was rejected approximately 50% of the time by the ANCOVA analysis of the same data, despite the fact that Figure 1B indicates that the average listening comprehension score is the same for native and non-native speakers. Hence, applying an ANCOVA to these data leads to a serious elevation of the Type I error rate when evaluating the main effect due to a between-subjects factor.

Figure 1C, indicates a different situation where an absolute difference of 3.2 units is introduced between the mean listening comprehension scores in the two groups (μ_*L, non−native speakers*_ = 48.4, μ_*L, native speakers*_ = 51.6), with all other parameters remaining the same. In this example, the research hypothesis concerning the Group effect is true, namely that there is a main effect of Group (*B* ≠ 0, there is a difference between the native and non-native speakers in terms of performance on the listening comprehension task). Yet an ANCOVA conducted on simulations based on these distributions (with 40 points in each group) rejected the null hypothesis that there was no main effect due to group only approximately 5% of the time, whereas this null hypothesis was rejected approximately 56% of the time in the corresponding ANOVA analysis. This result follows directly from an examination of the expected value of the mean square for the main effect when there is a difference between the covariate population means in the two groups (see Table 1B), because in the general linear model of these data, the slope parameter is α = 0.4, and the parameter for the Group effect is *B* = −1.6 for the non-native speakers, so that 2 B − α (μ_*x*, *EL2*_ − μ_*x*, *EL1*_) = −3.2 − 0.4 (17–25) = 0. Hence an ANCOVA of data generated by this model fails to detect a difference between the means of the dependent variable of the magnitude shown in Figure 1C whereas the power to detect a difference of this magnitude is 0.56 for an ANOVA of the same data.

Figure 1D depicts a situation in which the expected value of the covariate measure is the same in both groups (μ_*x*1_ = μ_*x*2_ = 21) but there is still a difference of 3.2 between the listening comprehension measures in the two groups. Simulations in this case show that the power to reject the null hypothesis for the Group effect, when this specific research hypothesis is true, is greater for the ANCOVA analysis (*p* ≈ 0.62) than it is for the ANOVA analysis of the same data (*p* ≈ 0.56). This illustrates that when the expected values of the covariate are the same in both groups, and the covariate measure is correlated with the dependent variable, an ANCOVA provides a more powerful test of whether or not there is a main effect due to Groups than does an ANOVA.

An examination of Table 1 also indicates that an ANCOVA provides a valid test of the null hypothesis that α = 0, independent of the difference between the means of the covariate in the two groups. Hence this null hypothesis was rejected approximately 96% of the time for the three models depicted in Figures 1B–D. These example indicate that an ANCOVA always provides a valid test of the null hypothesis that the relationship of the covariate to the dependent variable is zero, but only provides a valid test of whether the means of the two groups with respect to the dependent variable differ from one another when the population mean values of the covariate measures are the same in both groups.

## Evaluating within-subject effects

The first thing to note is that when an ANCOVA is conducted on data collected in an experiment in which there are within-subject factors, the covariate measures must be centered across all of the participants in the experiment. When the experimental or classification design consists only of between-subjects factors, one does not need to worry about centering the covariate measures when using standard statistical packages because these packages automatically center the covariates^5^. However, when the experiment contains within-subject factors, these standard programs do not automatically center the covariate measures, and the user must do so before entering the measures into these programs. Although the need to center the covariate has been noted previously (e.g., Delaney and Maxwell, 1981) automatic centering of the covariate has not been incorporated into standard statistical packages such as SPSS, SAS, or R. Moreover, we are not aware of any mention of the need to center the covariate before entering data into these programs in any of the manuals that have been published for users of these three packages that we have examined^6^. Hence, when the experiment contains within-subject factors, it is necessary to center the covariates across all participants before using any of these programs.

Once the covariate measures have been centered, an ANCOVA applied to an experiment with within-subject factors can be quite useful under certain conditions. To illustrate the possible benefits of an ANCOVA in such a situation, we will examine the general linear model for an ANCOVA in a single-factor, within-subject design with only two levels of the within-subject factor. The model for such a design is:

(2) $$\begin{array}{l} y_{1,k}\text{​​}=\mu +W+\left(\alpha +\alpha_{d}\right)\left(x_{k}-\mu_{x}\right)+S_{k}+e_{ws1,k}\\ y_{2,k}\text{​​​}=\mu -W+\left(\alpha -\alpha_{d}\right)\left(x_{k}-\mu_{x}\right)+S_{k}+e_{ws2,k} \end{array}$$

where μ is the grand mean in the population, *W* is the effect due to being in level 1 of the within-subject factor, *S_k_* is the effect due to being subject *k* (1 ≤ *k* ≤ *n*), *x_k_* is covariate measure on subject *S_k_*, α is the coefficient specifying the average contribution of the covariate to the dependent variable, α_*d*_ specifies the extent to which the contribution of the covariate in level 1 of the within-subject factor differs from its average contribution to the dependent variable, and *e*_*ws*1,*k*_ and *e*_*ws*2,*k*_ are normally distributed random deviates whose mean is zero and whose standard deviation, σ_*ws*_, is the same for all subjects and levels of the within-subject factor. The covariate measure, *x_k_*, is also assumed to have a normal distribution in the population with a mean of μ_*x*_ and a standard deviation of σ_*x*_.

Note that this ANCOVA model allows the linear relationship between the covariate and the dependent variable to differ for different levels of the within-subject factor. Specifically, the slope of the function relating the dependent variable in level 1 of the within-subject factor to the covariate measure is α + α_*d*_, whereas in level 2, the slope is α − α_*d*_. Hence, the slope difference between the two levels of the within-subject factor is 2α_*d*_. Indeed, one of the advantages of conducting an ANCOVA in a within-subject design is that one can test whether the slope of the line relating the dependent variable to the covariate is altered by the different levels of the within-subject factor by testing the null hypothesis that α_*d*_ = 0.

The Table 2A presents the expected values of the mean squares for an ANCOVA for a single-factor, within-subject design with two levels when the covariate has been centered before submitting the data to one of the standard statistical packages. Table 2 shows that an ANCOVA successfully removes any contribution of the covariate to the mean square error and provides a valid test of the null hypothesis that α_*d*_ = 0. However, it does not provide a valid test as to whether the difference between the two conditions is significant because the mean square for the within-subject main effect is contaminated by the presence of covariant variance, whereas the mean square error is not. Hence, the probability of a Type 1 error will be elevated when α_*d*_ ≠ 0. To evaluate the main effect of the within-subject factor, one needs to conduct an ANOVA on the data (see Table 2B). Hence, when analyzing data in a design that is solely within-subject, once the measures have been centered, one can use an ANCOVA to estimate the within^*^covariate interaction, but then should employ a standard ANOVA to evaluate any effects *not* involving the covariate.

**Table 2:** (A) Expected values for an ANCOVA of a Within-Subject Experiment with two levels when the covariate measures are centered for the model described in Equation (2). W^*^C is the interaction between the within-subject factor and the covariate. Before conducting an ANCOVA with standard statistical packages, be sure to center the covariate. **(B)** Use an ANOVA to estimate all Within-Subject Sources of Variance other than that due to the interaction between the Within-Subject factor and the Covariate.

**(A)**			
**Source**	***df***	**ANCOVA: E[Mean Square] when the covariate measures are centered**	***F***
Within	1	2*nW*^2^ + 2α^2^_*d*_σ^2^_*x*_ + σ^2^_*ws*_	$\frac{MS_{Within}}{MS_{Error}}$ is not a valid test of *H*0: *W* = 0
W^*^C	1	2(*n* − 1)α^2^_*d*_σ^2^_x_ + σ^2^_*ws*_	$\frac{MS_{W*C}}{MS_{Error}}$ is a valid test of *H*0: α_*d*_ = 0
Error	n−2	σ^2^_*ws*_	
**(B)**			
**Source**	***df***	**ANOVA: E[Mean Square] when the covariate measures are centered**	***F***
Within	1	2*nW*^2^ + 2α^2^_*d*_σ^2^_*x*_ + σ^2^_*ws*_	$\frac{MS_{Within}}{MS_{Error}}$ is a valid test of *H*0: *W* = 0
Error	n−1	2α^2^_*d*_σ^2^_x_ + σ^2^_*ws*_	

## Mixed between-subjects and within-subject design

We have seen that an ANCOVA of a between-subjects design provides valid tests of all between-subjects effects when the following two assumptions are met: Assumption 1, the slope of the line relating the covariate to the dependent variable is the same for all levels of the between-subjects factor, and Assumption 2, the expected value of the covariate in each level of the between-subjects factor is the same. Recall that the latter assumption will be met if subjects are randomly assigned to the different levels of the between-subjects factor but is unlikely to be met when the different levels represent different populations of subjects. We have also seen that when the design includes only within-subject factors, an ANCOVA can be used to test for interactions between the within-subject factors and the covariate, but an ANOVA should be used for evaluating all other within-subject effects. Hence, in a mixed between-subjects and within-subject design, all tests in the between-subjects portion of the analysis will be valid when Assumptions 1 and 2 are met, as well as any interaction between the covariate and within-subject factors in the within-subject portion of the ANCOVA as long as the covariate is centered by the experimenter. However, this does not address the question of whether or not the tests involving interactions between within-subject and between-subjects factors provided by an ANCOVA are valid even when the two above-mentioned assumptions are met.

To evaluate how Between^*^Within interactions are handled in an ANCOVA, we have examined what happens to the Between^*^Within interaction in a mixed model with within- and between-subjects factors with two levels each. The equations defining this model are:

(3) $$\begin{array}{l} y_{1,1,k}\text{​​​}=\mu +B+W+BW+\left(\alpha +\alpha_{d}\right)\left(x_{1,k}-\mu_{x1}\right)\\ \;+S_{1,k}+e_{ws1,1,k}\\ y_{1,2,k}\text{​​​}=\mu +B-W-BW+\left(\alpha -\alpha_{d}\right)\left(x_{1,k}-\mu_{x1}\right)\\ \;\;+\;S_{1,k}+e_{ws1,2,k}\\ y_{2,1,k}\text{​​}=\mu -B+W-BW+\left(\alpha +\alpha_{d}\right)\left(x_{2,k}-\mu_{x2}\right)\\ \;+\;S_{2,k}+e_{ws2,1,k}\\ y_{2,2,k}\text{​​​}=\mu -B-W+BW+\left(\alpha -\alpha_{d}\right)\left(x_{2,k}-\mu_{x2}\right)\\ \;+\;S_{2,k}+e_{ws2,2,k} \end{array}$$

where the first subscript of *y* specifies the level of the between-subjects factor, the second subscript of *y* specifies the level of the within-subject factor, and *k*, *n*, μ, *B*, *W*, *x*_1,*k*_, *x*_2,*k*_, α, α_*d*_, μ_*x*1_, μ_*x*2_, *S*_1,*k*_, and *S*_2,*k*_ are as defined above. The Table 3A presents the expected values of the various within-subject sums of squares when Assumptions 1 and 2 apply in this mixed 2 × 2 design, and the covariate is centered by the experimenter. An examination of this table shows that when these assumptions are met, the within-subject section of the ANCOVA removes the source of variance due to the covariate in the error term, and provides a valid test of the Within^*^Covariate interaction (i.e., a valid test of the null hypothesis that α_*d*_ = 0), and a valid test of the Between^*^Within interaction (i.e., a valid test of the null hypothesis that *BW* = 0). However, as an examination of the mean squares indicates, it does not provide a valid test of the within-subject main effect because the mean square for the within-subject main effect is contaminated by the variability in the covariate when α_*d*_ ≠ 0 whereas the mean square error is not. Finally, because the between-subjects portion of the general linear model is based on the average performance of a participant (averaged over within-subject effects), all tests involving between-subjects factors in an ANCOVA will also be valid when the data satisfy Assumptions 1 and 2, and the covariate measures are centered by an experimenter.

**Table 3:** (A) Expected values of the Mean Squares for the within portion of mixed 2 × 2 ANCOVA when μ_*d*_ = 0 (μ_*x*1_ = μ_*x*2_). W^*^C and W^*^B are the Within^*^Covariate and the Within^*^Between interactions, respectively. **(B)** The expected values of the Within portion of an ANCOVA when μ_*d*_ ≠ 0 (μ_*x*1_ ≠ μ_*x*2_). Note: *PDF_NCF_* is the non-central *F* distribution with *df*_1_ = 1, *df*_2_ = 2(*n* − 1), and non-centrality parameter, λ = (n μ^2^_*d*_)/(2 σ^2^_*x*_), where *n* is the number of participants in each group. **(C)** Expected Value and *F* test of the W^*^B interaction and within-subject main effect from the within section of an ANOVA.

**(A)**			
**Source**	***df***	**ANCOVA: E[Mean Square] when μ_*d*_ = 0**	***F***
Within	1	4*nW*^2^ + 2α^2^_*d*_σ^2^_*x*_ + σ^2^_*ws*_	$\frac{MS_{Within}}{MS_{Error}}$ is not a valid test of H0: *W* = 0
W^*^C	1	4(*n* − 1)α^2^_*d*_σ^2^_*x*_ + σ^2^_*ws*_	$\frac{MS_{W*C}}{MS_{Error}}$ is a valid test of H0: α_*d*_ = 0
W^*^B	1	$\frac{\text{8}n\left(n-1\right)BW^{2}}{2n-1}+\sigma_{ws}^{2}$	$\frac{MS_{W*B}}{MS_{Error}}$ is a valid test of H0: *BW* = 0
Error	2n–3	σ^2^_*ws*_	
**(B)**			
**Source**	***df***	**ANCOVA: E[Mean Square] when μ_*d*_ ≠ 0**	***F***
Within	1	4*nW*^2^ + 2α^2^_*d*_σ^2^_*x*_ + σ^2^_*ws*_	$\frac{MS_{Within}}{MS_{Error}}$ is not a valid test of H0: *W* = 0
W^*^C	1	4(*n* − 1)α^2^_*d*_σ^2^_*x*_ + σ^2^_*ws*_	$\frac{MS_{W*C}}{MS_{Error}}$ is a valid test of H0: α_*d*_ = 0
W^*^B	1	$\int_{x\;=\;0}^{\infty }\frac{2n\left(n-1\right){\left(2BW-\alpha_{d}\left(\mu_{x1}-\mu_{x2}\right)\right)}^{2}}{2(n-1)+f}PDF_{NCF}[df1,df2,\lambda ,f]df+\sigma_{ws}^{2}$	$\frac{MS_{W*B}}{MS_{Error}}$ is not a valid test of H0: *BW* = 0
Error	2n–3	σ^2^_*ws*_	
**(C)**			
**Source**	***df***	**ANOVA: E[Mean Square]**	***F***
Within	1	4*nW*^2^ + 2α^2^_*d*_σ^2^_*x*_ + σ^2^_*ws*_	$\frac{MS_{Within}}{MS_{Error}}$ is a valid test of H0: *W* = 0
W^*^B	1	4*nBW*^2^ + 2α^2^_*d*_σ^2^_*x*_ + σ^2^_*ws*_	$\frac{MS_{W*B}}{MS_{Error}}$ is a valid test of H0: *BW* = 0
Error	2n–2	2α^2^_*d*_σ^2^_*x*_ + σ^2^_*ws*_	

This raises the question of how to analyze the data from an experiment in which Assumption 2 is unlikely to be valid. Such is likely to be the case when the different levels of the between-subjects factor represent different populations of participants (e.g., musicians versus non-musicians, young versus old adults). The Table 3B presents the expected sums of squares of the within-subject effects in a mixed 2 × 2 design when Assumption 2 does not hold but where the experimenter has centered the covariate before submitting the data to a standard ANCOVA analysis. This Table 3B shows that the only test that is valid in the within-subject portion of the ANCOVA is the Within^*^Covariate interaction. Moreover, simulations, similar to those carried out for single factor, between-subjects designs (see Figure 1) indicate that substantial increases in Type 1 error rates, as well as substantial losses in power can occur in these designs when the means of the covariates differ in a classification design, and there are correlations among the dependent variable and the covariate. Hence, in this case, the appropriate solution is to use an ANOVA to evaluate all other within-subject effects. The Table 3C shows the expected values obtained from the within-subjects portion of an ANOVA of the data.

## How to use ANCOVA in mixed between-subjects and within-subject designs

When it is reasonable to assume that the expected value of a covariate measure is the same for each grouping of subjects, one can use a standard ANCOVA to analyze the data provided that one first centers the covariate before entering the data into a standard statistical package. If this is done an examination of the μ_*d*_ = 0 (μ_*x*1=_ μ_*x*2_) portion of Tables 1, 3 indicate that all *F*-tests involving the covariate, and all tests involving the between-subjects factor are not only valid, but also more precise because the ANCOVA eliminates the contribution of the covariate to performance when conducting statistical tests.

**Figure 2:**
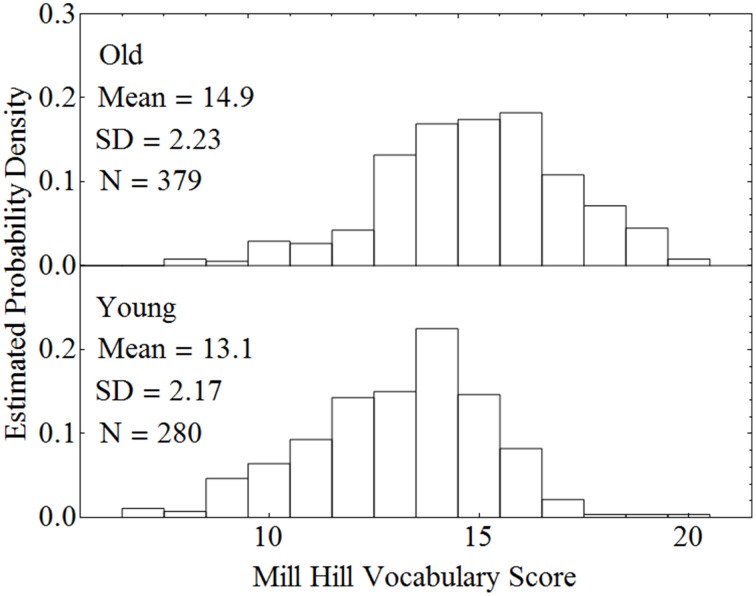
**Estimated probability density functions for older and younger adults on the Mill Hill Vocabulary test**.

**Figure 3:**
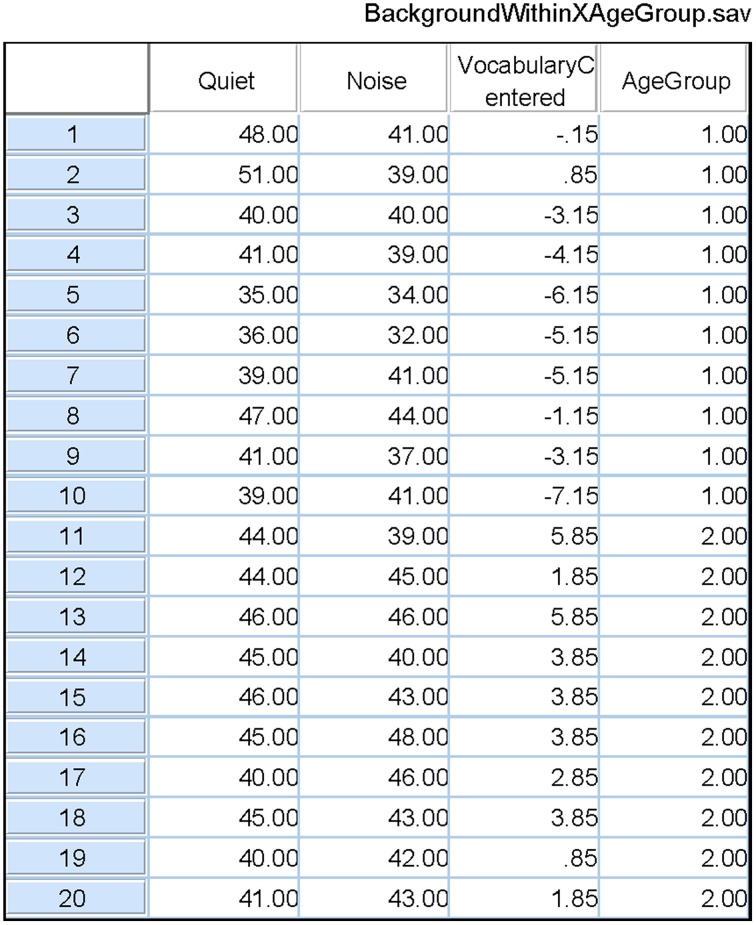
**The SPSS data file used as input to both an ANCOVA and an ANOVA of the data from Table 4**. Quiet and Noise are identified as the two levels of the Within-Subject factor in a repeated measures analysis. Age Group is the Between-Subjects factor in this analysis. In the ANCOVA, the covariate is the Centered Vocabulary scores. The output of these analyses are shown in Table 5.

When there is reason to believe that the expected values of the covariate measures in the two groups are substantially different, conducting a standard ANCOVA can lead to serious errors, and a different procedure should be followed. Consider, for example, a classification design in which the experimenter wishes to compare younger and older adults with respect to how well they can comprehend spoken material in different levels of background noise. Two age groups constitute the between-subjects factor. Let the within-subject factor be the level of a background masker (quiet versus steady-state noise), and the covariate be vocabulary size. Data from our laboratory indicate that older adults typically have a larger vocabulary score than younger adults. Figure 2 plots estimated probability density functions for Mill Hill Vocabulary scores based on data collected in our lab over the past few years for two age ranges (young, 17–32, *M* = 21; old, 60–91, *M* = 73). Figure 2 shows that these scores are normally distributed with the same variance in both age groups. Indeed an *F*-test of the ratio of sample variances failed to reject the null hypothesis that the two population variances were equal [*F*_(379, 280)_ = 1.06, *p* > 0.5]. However, a *t*-test of the hypothesis that the population means were equal clearly indicates that they are not [*t*_(657)_ = 10.52, *p* < 10^−20^]. Hence, the expected value of this covariate is not likely to be the same in both age groups and it is best to assume that μ_*d*_ ≠ 0. Sample data for the above specified design are presented in Table 4. In such a case, the recommended procedure is to conduct a standard ANCOVA to test hypotheses concerning the effect of covariate (α) and the Within^*^Covariate interaction (α_*d*_), and to use a standard repeated measures ANOVA (without the covariate) to evaluate all other effects. Figure 3 shows the data file that served as input to SPSS (version 22). Both an ANCOVA and an ANOVA were performed on the data. Table 5 presents the hybrid analysis for this type of design for the input shown in Figure 3. In this hybrid analysis, the only test taken from the Within Section of the ANCOVA is the Within^*^Covariate interaction (Background^*^VocabularyCentered), and the only test taken from the Between Section of the ANCOVA is main effect of the Covariate (VocabularyCentered). The tests of the main effect due to the Within factor (Background) and the Within^*^Between interaction (Background^*^AgeGroup) are taken from the Within Section of the ANOVA, and the main effect of the Between factor (AgeGroup) is taken from the Between Section of the ANOVA.

**Table 4:** **Hypothetical number of questions correctly answered under two different levels of background noise (Quiet vs. Noise, within-subject factor) by subjects sampled from two different age groups (Young vs. Old, between-subjects factor)**.

**Subject no**.	**Age group**	**Background noise level**		**Covariate (vocabulary size)**	**Covariate (centered)**	**Covariate (centered within each group)**
		**Quiet**	**Noise**			
1	Young	48	41	17	−0.15	3.3
2	Young	51	39	18	0.85	4.3
3	Young	40	40	14	−3.15	0.3
4	Young	41	39	13	−4.15	−0.7
5	Young	35	34	11	−6.15	−2.7
6	Young	36	32	12	−5.15	−1.7
7	Young	39	41	12	−5.15	−1.7
8	Young	47	44	16	−1.15	2.3
9	Young	41	37	14	−3.15	0.3
10	Young	39	41	10	−7.15	−3.7
11	Old	44	39	23	5.85	2.4
12	Old	44	45	19	1.85	−1.6
13	Old	46	46	23	5.85	2.4
14	Old	45	40	21	3.85	0.4
15	Old	46	43	21	3.85	0.4
16	Old	45	48	21	3.85	0.4
17	Old	40	46	20	2.85	−0.6
18	Old	45	43	21	3.85	0.4
19	Old	40	42	18	0.85	−2.6
20	Old	41	43	19	1.85	−1.6

**Figure 4:**
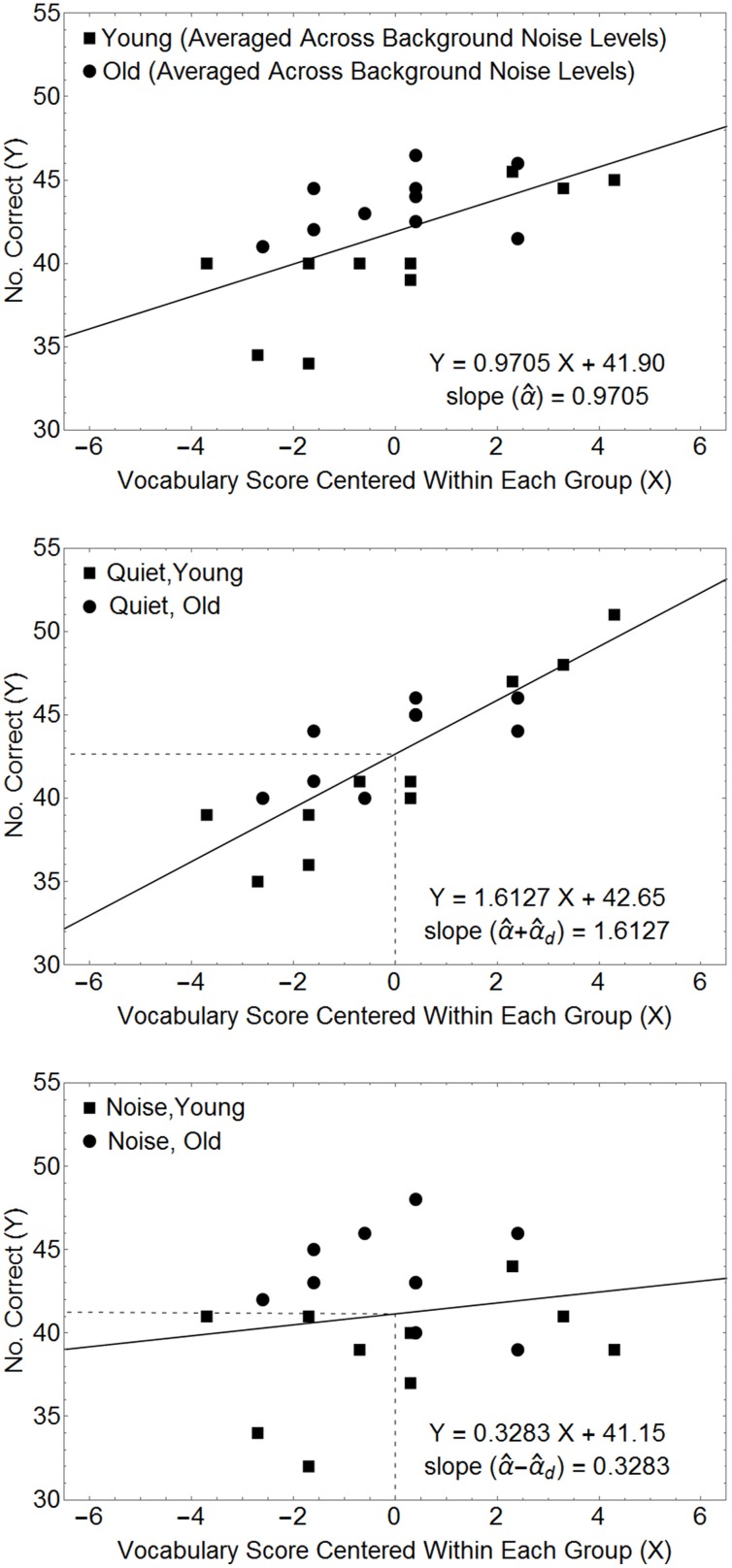
**Relationships between the number of questions answered correctly and the covariate (centered in each age group) for the data in Table 4**. The top panel plots the number of questions answered correctly, averaged over the within-subject factor, as a function of the covariate measures. The middle and bottom panels plot the data for the quiet and noisy conditions. The estimated scale factors for the different conditions ($\hat{\alpha }$, $\hat{\alpha }$_*d*_) can be obtained from the slopes of the lines in these plots. In this model the estimated within-subject difference (*ŵ*_1_ − *ŵ*_2_) is the difference between the intercepts of the two straight lines in the lower two panels. Hence, in this example, the Mean Square for the Within-Subject Main Effect is 10 × (42.65 − 41.15)^2^ = 22.5, as computed by the ANOVA.

The covariate measure is vocabulary size. To center the covariate measures across groups, compute the mean value of the covariate for all of the subjects and subtract this value from each of the covariate measures. This is how the column labeled “Covariate (Centered)” was obtained. It is the centered covariate measures that are entered into the analyses (see Figure 3). In the column labeled “Covariate (Centered within each group),” the mean of the covariates in each group is subtracted from the covariate measures in that group. The covariate measures centered within each group are not entered as input to the statistical package. However, they are useful in interpreting the results (see Figure 4).

**Table 5:** **Composite ANCOVA table for the Table 4 data**.

**Source**	**Type III Sum of Squares**	***df***	**Mean Square**	***F***	**Sig**
**TESTS OF WITHIN-SUBJECT EFFECTS**					
Background*VocabularyCentered (from ANCOVA)	71.348	1	71.348	17.693	0.001
Error term (from ANCOVA)	68.552	17	4.032		
Background (from ANOVA)	22.500	1	22.500	2.895	0.106
Background*AgeGroup (from ANOVA)	19.600	1	19.600	2.522	0.130
Error (from ANOVA)	139.900	18	7.772		
**TESTS OF BETWEEN-SUBJECTS EFFECTS**					
VocabularyCentered (from ANCOVA)	162.950	1	162.950	15.076	0.001
Error (from ANCOVA)	183.750	17	10.809		
AgeGroup (from ANOVA)	108.900	1	108.900	5.654	0.029
Error (from ANOVA)	346.700	18	19.261		

The data were submitted first to a repeated measures ANCOVA with Vocabulary as a Covariate. Note that the Vocabulary scores were centered when they were submitted to the ANCOVA. The Within*Covariate and the Main effect of the covariate are evaluated within the ANCOVA. All other effects are taken from an ANOVA on the same data without the covariate.

## Interpretation of interactions between covariates and within-subject factors

The Within^*^Covariate interaction term tests whether the slope of the line relating the covariate to the dependent measure differs among the different levels of the within-subject factor. Figure 4 plots the relationship between the dependent variable and the covariate (centered within each age group) for the example used above to visualize the contribution of the covariate to the average performance of each subject (top panel), and to the different levels of the within-subjects factor (middle and lower panels). Here we find that the contribution of the covariate to performance is lessened in a noisier environment. This would be consistent with a hypothesis that the presence of noise disrupts lexical processing. Because this model specifically hypothesizes that the expected value of the covariate differs between the two groups, it is reasonable to estimate the level of difference between the two functions relating the dependent variable to the covariate as the difference between these two functions at the point on the abscissa that represents our best estimate of the expected values of each of the covariate measures in each group. Note that our best estimate of the population mean covariate in each group occurs when *x*_*i,k*_ = *x*_*i*_. Hence, the difference in the intercept values of the two linear functions in the two lower panels of Figure 3, provides an unbiased estimate of 2*W*.

In general, when there is reason to believe that the population mean value of the covariate is the same across all subject groups, the data can then be submitted to a standard ANCOVA package provided that the covariate measures are centered across all subjects before entering the data into a standard statistical package (centering the covariate is a necessary step when the design contains within-subject factors). If this is done then all of the tests involving the covariate and all of the tests involving between-subjects factors in both the Within-Subject and Between-Subjects portion of the ANCOVA will be valid. The remaining within-subject effects then should be evaluated using an ANOVA. When there is some doubt as to whether the population mean covariate is the same across all groups, conduct both an ANCOVA and an ANOVA. Use the ANCOVA for testing the main effect of the covariate and the Within^*^Covariate interaction. Then use the ANOVA to test all other remaining effects. Table 6 specifies the recommended steps to be followed when: (1) all factors are within-subject; (2) the design contains between-subjects factors where the expected value of the covariate is the same for all groups of subjects; and (3) the expected value of the covariate might differ across groups.

**Table 6:** **Recommended procedures to follow when conducting an ANCOVA for three types of designs: (1) All factors are Within-Subject; (2) Experimental designs in which subjects are randomly selected from a uniform population and randomly assigned to different experimental conditions, and (3) Classification designs in which the different levels of Between-Subjects factor consist of samples from different populations (e.g., musicians and non-musicians) where it cannot be assumed the expected value of the covariate is the same across populations**.

**All factors Within-Subject**	**Experimental, Between-Subjects Designs with or without a Within-Subject component (subjects randomly selected from a uniform population and randomly assigned to different experimental conditions)**	**Classification Designs (with or without a Within-Subject component) where it cannot be assumed that the expected value of the covariate measures is the same for each group of participants (e.g., the different levels of the Between-Subject factor represent random samples from different populations)**
1. Center the covariate measures	1. Center the covariate measures^*^	1. Center the covariate measures
2. Conduct an ANCOVA	2. Conduct an ANCOVA	2. Conduct an ANCOVA
3. Use the ANCOVA to evaluate all effects involving covariates	3. Use the ANCOVA to evaluate all Between-Subjects effects and any interactions of Between-Subjects and Within-Subject effects, including Within^*^Covariate interactions	3. Use the ANCOVA to evaluate all effects involving a covariate
4. Conduct an ANOVA	4. Conduct an ANOVA	4. Conduct an ANOVA
5. Use an ANOVA to evaluate all remaining effects	5. Use an ANOVA to evaluate all remaining Within-Subject effects	5. Use the ANOVA to evaluate all remaining effects

*Note that whenever between-subject factors are involved, it is important to first test whether the relationship between the dependent variable and the covariate is the same for all levels of the between-subjects factor (e.g., Howell, 2010, p. 600–603)*.

* *Although it is not necessary to center the covariate measures before entering the data into a standard statistical package when all factors are Between-Subjects, it is necessary to do so when the experimental design contains Within-Subject factors because these programs do not center the covariate measures when evaluating within-subject effects. To be safe, always center the covariate measures before entering them into a statistical package*.

## Concluding remarks

In psychological research, we often have reason to believe that two different measures taken on individuals are likely to be correlated in the population from which individuals were sampled. For instance, we would expect measures of listening comprehension to be correlated with measures of reading comprehension because a common set of linguistic and cognitive processes are likely to be engaged when information is received either aurally or visually. Hence, the appropriate sampling model, given that both measures are normally distributed, is one in which paired observations are being sampled from bi-normal distributions like those shown in Figure 1. If one of the two measures is the main variable of interest, it would appear to be sensible to enter the other measure as a covariate. When the expected value of the covariate measure is the same in every group of subjects in a between-subjects design, conducting an ANCOVA reduces both the error sum of squares, and the sum of squares due to the Group main effect, thereby increasing the power of tests involving group differences. Note that this is a reasonable assumption in experimental designs, in which subjects are drawn from the same population and are randomly assigned to different levels of the between-subjects factor.

However, the ANCOVA in classification designs, where the different levels of a between-subjects factor consist of individuals sampled from different populations, is not so straightforward^7^. In such instances, tests involving between-subjects factors are contaminated by the differences among the expected values of the covariate measures across the different populations in the experimental design. In this paper, we have shown that the hybrid procedure, outlined in the third column of Table 6, circumvents these problems, and provides valid tests of all of the parameters of the model.

In conclusion, we urge investigators, who have used SPSS or any equivalent package to conduct an ANCOVA in designs which contained one or more within-subject factors (repeated measures designs), to re-examine their analyses to see if and how the covariate or covariates were centered before performing the ANCOVA. If the covariate measures were not centered in designs involving within-subject factors before entering the data into these packages, the data should be reanalyzed with the measures centered across all subjects. If between-subject factors were included in the design, and it is reasonable to expect that there might be differences in the expected values of the covariate measures across different groupings of subjects, the data should be re-analyzed following the procedures recommended. Alternatively, one should look for another means of analyzing the data, which take into account model assumptions, and the nature of the experimental design and the questions to be asked.

### Conflict of interest statement

The authors declare that the research was conducted in the absence of any commercial or financial relationships that could be construed as a potential conflict of interest.

## Footnotes

^1^Although a number of authors (e.g., Anderson, 1963; Huitema, 1980; Algina, 1982; Howell, 2010) have noted that violation of the assumption that the expected values of the covariate are the same for all groups can lead to serious issues with respect to the interpretation an ANCOVA, to our knowledge, there has been no thorough exploration of how such violations affect the interpretation of results and the validity of statistical tests.

^2^In Section A2 of the Appendix (see Supplementary Material) we show that Equation (1) specifies the general linear model that is appropriate for a situation in which: (1) the dependent variable and the covariate are both normally distributed in each of the two groups; and (2) the correlation between the dependent variable and the covariate is the same in both groups.

^3^The derivation of the expected values of this and the other designs considered here are to be found in Section B of the Appendix (see Supplementary Material).

^4^The expected value of *e*_*x*,1_ = the expected value of *e*_*x*,2_ = 0, and the expected value of (*x*_1,*k*_ − μ_*x*1_) = the expected value of (*x*_2,*k*_ − μ_*x*2_) = 0, so that the expected value of *y*_1,*k*_ = μ + *B*, and the expected value of *y*_2,*k*_ = μ − *B*.

^5^To confirm that the covariates are centered by the standard statistical packages when there are only Between-Subjects factors, conduct such an analysis with and without the covariates centered. The results of the analysis will be the same in both instances. However, if the design contains only Within-Subject factors, the sums of squares for Within-Subject effects that do not involve the covariate will differ depending upon whether or not the covariates are centered. Since it is also true that these packages do not center the covariate in mixed Within-Subject and Between-Subjects designs, the experimenter should center the covariate measures whenever the design has Within-Subject factors.

^6^We could find no mention of this problem in three commonly used SPSS manuals: (1) Bryman and Cramer (2009), (2) Field (2009), and (3) Norušis (2004), and we could not find any clear warning to center the covariate in within-subject designs in two SAS manuals: (1) Der and Everitt (2009), and Marasinghe and Kennedy (2008). The R manuals we reviewed (Faraway, 2005; Gries, 2009; Pace, 2012) did not discuss the use of ANCOVA in within-subject or mixed within-subject and between-subjects designs.

^7^It is interesting to note that the division of a population into different subgroups leads to interpretational difficulties in other designs. These include the paradoxical results (Yule, 1903; Simpson, 1951) that can occur in the analysis of contingencies among dichotomous variables; or how the correlation between before and after measures of an attribute clouds the interpretation of whether or not the passage of time has a differential effect on different populations of subjects (Lord, 1967). For a discussion of the interpretational difficulties inherent in these designs and possible ways to resolve them, see Tu et al. (2008).
