# Supplementary material for: A cautionary note on the use of the Analysis of Covariance (ANCOVA) in classification designs with and without within-subject factors
Source: Front Psychol. 2015 Apr 21;6:474. doi: 10.3389/fpsyg.2015.00474 (PMC4404726; doi:10.3389/fpsyg.2015.00474)
Supplement: Supplementary file 1 [file DataSheet1.DOCX]

**APPENDIX**

The canonical way of modeling correlations among a set of measures is to first assume that each measure is normally distributed and that the various measures may or may not be correlated. We refer to this model as the multi-normal model. In the case where there are only two variables, it is referred to as the bi-normal model. This model is completely characterized by two sets of parameters. The first set of parameters is the set containing the means or expected values (E) of each of the variables. The second is the covariance matrix which specifies the variances of each of the variables along the diagonal, and the covariances (relationships) among them in the off-diagonal elements. This matrix is symmetric and positive definite.

When referring to the within-subject and between-subjects variances in this multi-normal model we use the symbol $\sigma_{wsm}^{2}$ to refer to the within-subject variance, and the symbol $\sigma_{bsm}^{2}$to refer to the between-subjects variance, where the letter *m* appended to the subscript *ws* or *bs* specifies that the variance in question is specific to the multi-normal model and differs from the within-subject ($\sigma_{ws}^{2}$) and between-subjects ($\sigma_{bs}^{2}$) variance in the general linear model. In some cases the within-subject variance in the multi-normal model is the same as in the general linear model. In that event, the within-subject variance is referred to as $\sigma_{ws}^{2}$ in both models.

The Appendix is divided into two sections. In Section A, we will first show that there is a one-to-one correspondence between the multi-normal model of covariance and the general linear model of the same data. We will do this by specifying the covariance matrix in each model, and then showing that there is an isomorphic way of mapping the covariance matrix associated with the multi-normal model into the covariance matrix of the general linear model of the same set of data, and vice versa. Demonstrating this correspondence requires relating the correlation (*ρ*) between two variables in the multi-normal model to the slope of the line (*α*) relating one of the variables to the other in the general linear model. It also requires the specification of how within-subject and between-subjects variances are related in the two models. When we refer to within-subject variance in the general linear model, we will use the symbol $\sigma_{ws}^{2}.$When referring to between-subjects variance in the general linear model, we will use the symbol $\sigma_{bs}^{2}$. In Section A we will show that we can define α,$\sigma_{ws}^{2}$ and $\sigma_{bs}^{2}$ in the general linear model in terms of *ρ*,$\sigma_{wsm}^{2}$ and $\sigma_{bsm}^{2}$ from the multi-normal model in a way that produces equivalent covariance matrices in the two models, thereby establishing that they are isomorphic. We will begin by linking the multi-normal model to the general linear model for a single group. This will be followed by linking the multi-normal model to the general linear model when two groups are involved. We will then turn to a single-group design with two levels of a within-subject factor, followed by a two-group design with two levels of a within-subject factor, and show that the multi-normal model is equivalent to the general linear model of the same data.

Note also that when specifying parameters of the multi-normal and regression model, we will distinguish between latent variables and observable variables. For instance, individual variations among subjects are not directly observable in the models considered here because they always occur in the presence of within-subject variation. Hence, the subscript identifying the groups associated with such subject effects will contain the letter *L*, to indicate that this is a latent variable. For example ${SL}_{1,k}$ refers to the effect associated with subject *k* in group 1.

In Section B we will derive the expected values of the sums of squares in each of these designs in terms of the parameters of the general linear model.

**A. Determining the appropriate linear model to represent covariances among normally distributed variables**

**A1. Covariance within a Single Group**

In a single group design, the dependent measure taken on *k*th subject*,* $y_{k}$, is considered to be a joint function of the effect associated with being the *k*th subject, and the contribution of within-subject variability. We make the usual assumptions that: 1) within-subject variability is normally distributed and the same for all subjects; 2) the subject effect is also normally distributed; and 3) the effect due to the subject and the effect due to within-subject variability are independent of each other. Note that the effects due to the subject and to within-subject variability are not directly observable. Hence we will refer to them as latent variables, and represent the effect of being subject *k* by the symbol, ${SLm}_{k}$, and the effect of within-subject variability on the *k*th subject by the symbol, ${WL}_{k}$, where *L* specifies that it is a latent variable, *m* indicates that the nature of this latent variable differs from that of the corresponding latent variable in the general linear model.^[[1]](#footnote-1)^ The mean values of the ${WL}_{k}$ and ${SLm}_{k}$ random normal variables are 0 and *μ*, respectively, and their standard deviations are $\sigma_{ws} \mathrm{and} \sigma_{bsm}$, respectively. The letter *m* is appended to the subscript, *bs*, indicates that this between-subject variance is associated with the bi-normal model, and differs from the between-subject variance specified in the general linear model.

The covariate measure on the *k*th subject, $x_{k}$, is also assumed to be normally distributed with a mean of $\mu_{x}$ and a standard deviation of $\sigma_{x}$. The ${WL}_{k} and the {SLm}_{k}$ random normal variables are assumed to be independent of one another as are the $x_{k}$ and ${WL}_{k}$. Finally, we assume that the $x_{k}$ and the ${SLm}_{k}$ are correlated with the correlation coefficient between them being *ρ*. Hence the covariance matrix relating the $x_{k}, {WL}_{k}, and {SLm}_{k}$ normal variables is

$\left( \begin{matrix} \sigma_{x}^{2} & 0 & \rho\sigma_{x}\sigma_{bsm} \\ 0 & \sigma_{ws}^{2} & 0 \\ \rho\sigma_{x}\sigma_{bsm} & 0 & \sigma_{bsm}^{2} \end{matrix} \right)$ (A1)

Since an observable measure, $y_{k}$ represent the sum of two normally distributed and independent latent variables (${WL}_{k}$ and ${SLm}_{k}$), the covariance between the $y_{k}$ and $x_{k}$ measures becomes

$Cov\left[ y_{k}x_{k} \right]=Cov\left[ \left( {WL}_{k}+{SLm}_{k} \right)x_{k} \right]=Cov\left[ {WL}_{k}x_{k} \right]+Cov\left[ {SLm}_{k}x_{k} \right]= \rho\sigma_{x}\sigma_{bsm}$ (A2)

with the mean and variance of the $y_{k}$ measures being *μ* and $\sigma_{ws}^{2}$ + $\sigma_{bsm}^{2}$, respectively. Hence the covariance matrix relating the $x_{k}$ and $y_{k}$ variables in the bi-normal model becomes

$\left( \begin{matrix} \sigma_{x}^{2} & \rho\sigma_{x}\sigma_{bsm} \\ \rho\sigma_{x}\sigma_{bsm} & \sigma_{ws}^{2}+\sigma_{bsm}^{2} \end{matrix} \right)$ (A3)

The general linear model that is equivalent to this bi-normal model is

$y_{k}=\alpha\left( x_{k}-\mu_{x} \right)+{WL}_{k}+{SL}_{k}$ (A4)

where $y_{k}$ specifies the value of the dependent variable that is associated with the *k*th subject in the sample, $x_{k}$ and${WL}_{k}$ are the same variables as in the bi-normal model, and *α* is the coefficient relating the covariate to the independent variable.^[[2]](#footnote-2)^ The $x_{k}, {WL}_{k}$ and ${SL}_{k}$ are considered to be normally distributed independent random variables, with ${SL}_{k} \mathrm{and} {WL}_{k}$ being latent normal random variables since they cannot be observed directly, but only indirectly through their effects on $y_{k}$. The expected values of $x_{k}, {WL}_{k}$, and ${SL}_{k}$ are $\mu_{x}$, 0, and *μ*, respectively. Their variances are specified as $\sigma_{x}^{2}, \sigma_{ws}^{2}$, and $\sigma_{bs}^{2}.$ Note that the subscript denoting the variance of the ${SL}_{k}$ measures (*bs*) differs from the subscript (*bsm*) denoting the between-subject variance in the bi-normal model to indicate that the values of the between-subject variances differ between the two models. In the bi-normal model the covariate and the subject effect are correlated. In the general linear model the covariate and subject effect are assumed to be independent of one another, with the effect of covariate contributing in a linear fashion to the dependent variable.

The covariance of *x* and *y* in this general linear model is defined as

$$Cov\left[ y_{k},x_{k} \right]=Cov\left[ \left( \alpha\left( x_{k}-\mu_{k} \right)+{WL}_{k}+{SL}_{k} \right),x_{k} \right]$$

$Cov\left[ \alpha x_{k},x_{k} \right]-Cov\left[ \alpha\mu_{k},x_{k} \right]+Cov\left[ {WL}_{k},x_{k} \right]+Cov\left[ {SL}_{k},x_{k} \right]$ (A5)

$=\alpha\sigma_{x}^{2}$

The variance of the *y* values in the general linear model, given the mutual independence of ${WL}_{k},{SL}_{k}$, and $x_{k}$, are $\sigma_{y}^{2}=\sigma_{ws}^{2}+\sigma_{bs}^{2}+\alpha^{2}\sigma_{x}^{2}$. Therefore the covariance matrix for the general linear model is

$\left( \begin{matrix} \sigma_{x}^{2} & \alpha\sigma_{x}^{2} \\ \alpha\sigma_{x}^{2} & \sigma_{ws}^{2}+\sigma_{bs}^{2}+\alpha^{2}\sigma_{x}^{2} \end{matrix} \right)$ (A6)

The relationships of $\sigma_{bs}, \mathrm{and} \alpha$ to their corresponding parameters in the bi-normal model are:

$\sigma_{bs}=\sqrt{\sigma_{bsm}^{2}\left( 1-\rho^{2} \right)}, \mathrm{and} \alpha=\frac{\rho\sigma_{bsm}}{\sigma_{x}}$ (A7)

A substitution of the values in Equation A7 into Equation A6 shows that the covariance matrix for the general linear model is equivalent to the covariance matrix for the bi-normal model (Equation A3). Hence Equation A4 is the appropriate model to use in an ANCOVA of a single group design.

**A2. Covariance in a two-group between-subjects design**

The multi-normal model of a single factor, between-subjects design with 2 levels is specified by two bi-normal distributions, one for the first level of the between-subjects factor, and the other for the second level of the between-subjects factor. Since the two subject groups are tested separately, these two bi-normal distributions are independent of each other. The latent within-subject variables in the two groups (${\mathrm{the} WL}_{1,k} \mathrm{and} {WL}_{2,k}$) are assumed to have the same mean (0) and the same standard deviation ($\sigma_{ws}$). The means of the latent subject effects in the two groups, ${SLm}_{1,k} \mathrm{and} {SLm}_{2,k}$ are specified as *μ + B* and *μ - B*, respectively, with *B* specifying the additive effect of being in level 1 of the between-subjects factor. The variances of ${SLm}_{1,k} \mathrm{and} {SLm}_{2,k}$ are both assumed to be the same ($\sigma_{bsm}^{2}$). We will assume that the correlation coefficient, *ρ*, between the covariate measures, $x_{1,k}$, in group 1, and the subjects, ${SLm}_{1,k}$, in group 1 is the same as the correlation between the covariate measures, $x_{2,k}$, in group 2, and the subjects, ${SLm}_{2,k}$, in group 2. We also assume that the standard deviations of the $x_{1,k}$and $x_{2,k}$ are the same ($\sigma_{x}$). However, the expected values of the covariates in groups 1 and 2 ($\mu_{x1}$ and $\mu_{x2}$) are allowed to differ. Finally, we will assume that the ${WL}_{1,k}$,${WL}_{2,k}$, ${SLm}_{1,k}$, and ${SLm}_{2,k}$ variables are independent of one another as are the ${WL}_{1,k}$, ${WL}_{2,k}$, and $x_{k}$. Given these conditions, it follows that the covariance matrix for these three variables in group 1 ($x_{1,k}, {WL}_{1,k}$ and ${SLm}_{1,k}$) will be identical to that for the equivalent variables in group 2 ($x_{2,k}, {WL}_{2,k}$ and ${SLm}_{2,k}$), with both of them equal to the covariance matrix specified in Equation A1. It also follows that the covariance matrices for the observable variables in each of these two groups ($x_{1,k}$ and $y_{1,k}$ in group 1, and $x_{2,k}$ and $y_{2,k}$ in group 2) are both identical to that specified in Equation A3. The only difference between the two observable variables in both groups is that they have different means. Specifically, $y_{1},$ $y_{2}, x_{1}, \mathrm{and} x_{2}$ have the following distributions:

$y_{1}\sim N\left[ \mu+B,\sqrt{\sigma_{ws}^{2}+\sigma_{bsm}^{2}} \right], x_{1}\sim N\left[ \mu_{x1},\sigma_{x} \right]$

$y_{2}\sim N\left[ \mu-B,\sqrt{\sigma_{ws}^{2}+\sigma_{bsm}^{2}} \right], x_{2}\sim N\left[ \mu_{x2},\sigma_{x} \right]$ (A8)

The difference in means (2*B*) between the two levels of the dependent variable, *y*, reflects the additive effect of being in either level 1 or level 2 of the between-subjects factor. Different population means for the covariate might be expected if the two groups were sampled from different populations.

The general linear model that is equivalent to this multi-normal model is

$y_{1,k}=\alpha\left( x_{1,k}-\mu_{x1} \right)+{WL}_{1,k}+{SL}_{1,k}$

$y_{2,k}=\alpha\left( x_{2,k}-\mu_{x2} \right)+{WL}_{2,k}+{SL}_{2,k}$ (A9)

Note that the covariance matrices for both $y_{1,k}, \mathrm{and} y_{2,k}$ in this general linear model^[[3]](#footnote-3)^ are identical to that specified in Equation A6 and the relationship between the parameters of the general linear model to those of the multi-normal model are the same as those in Equation A7. Hence both the multi-normal and general linear models of this design have equivalent covariance matrices. It should also be clear that the arguments presented here can be extrapolated to more than one level of the between-subjects factor, and be generalized to characterize between-subjects designs with more than one factor. Hence from this point on, we will use the general linear model to ascertain the degree of covariance in the population, and to determine the expected values of the sums of squares calculated in an ANCOVA analysis of data when there is only a single between-subjects factor with two levels.

**A3. A single-group, Within-Subject Design with two levels of the Within-Subject factor**

Now consider a single group design in which multiple measures are taken on each subject. When there are only two levels in a single group, within-subject design, there are three observable measures: $x_{k}$ (the covariate measure associated with subject *k*), $y_{1,k}$, the *y* value for subject *k* tested in level 1 of the within-subject factor; and $y_{2,k}$, the *y* value for subject *k* tested in level 2 of the within-subject factor. We will begin by specifying the multi-normal model for the within-subject case in terms of three latent variables (${WLm}_{1,k}, {WLm}_{2,k}, \mathrm{and} {SLm}_{k})$ and one observable variable, the covariate measure ($x_{k}$). Again, *L* indicates a latent variable, and an *m* indicates that the parameters of this term differ between the multi-normal model and the regression model. These four variables (${x_{k}, WLm}_{1,k}, {WLm}_{2,k},$ and ${SLm}_{k})$ are all assumed to be normally distributed with populations means of $\mu_{x}, W, -W$ and *μ*, and standard deviations of ${\sigma_{x}, \sigma}_{wsm}, \sigma_{wsm}$ and $\sigma_{bsm}$ for ${x_{k}, WLm}_{1,k}, {WLm}_{2,k}$ and ${SLm}_{k}$, respectively. Again, the letter *m* appended to a subscript indicates that the value of the variable in question differs from its corresponding value in the general linear model. The correlation in the population between ${SLm}_{k}$ and the covariate is specified as *ρ* so that the covariance between the two measures is $\rho\sigma_{x}\sigma_{bsm}.$ The change in correlation between a subject’s performance and the covariate in within-subject Condition 1 is specified as $\rho_{d}$, and as $-\rho_{d}$in within-subject Condition 2. Therefore, the covariance between the ${WLm}_{1,k}$and $x_{k}$ is $\rho_{d}\sigma_{x}\sigma_{wsm}$, with the covariance between the ${WLm}_{2,k}$and the $x_{k}$ being -$\rho_{d}\sigma_{x}\sigma_{wsm}$. Because the ${WLm}_{1,k}$ and ${WLm}_{2,k}$are independent random variables, the correlation between them is 0. Finally, because both the ${WLm}_{1,k}$ and the ${SLm}_{k}$ are correlated with the $x_{k}$, the covariance between the ${WLm}_{1,k}$ and the ${SLm}_{k}$ is $\rho_{d}\rho\sigma_{wsm}\sigma_{bsm}$and the covariance between the ${WLm}_{2,k}$ and the ${SLm}_{k}$ is -$\rho_{d}\rho\sigma_{wsm}\sigma_{bsm}$. Therefore the covariance matrix for these four variables ($x_{k}, {WLm}_{1,k}, {WLm}_{2k}$, and ${SLm}_{k}$ ) is

$\left( \begin{matrix} \begin{matrix} \sigma_{x}^{2} & \rho_{d}\sigma_{x}\sigma_{wsm} \\ \rho_{d}\sigma_{x} \sigma_{wsm} & \sigma_{wsm}^{2} \end{matrix} & \begin{matrix} -\rho_{d}\sigma_{x}\sigma_{wsm} & \rho\sigma_{x}\sigma_{bsm} \\ 0 & \rho_{d} \rho\sigma_{wsm}\sigma_{bsm} \end{matrix} \\ \begin{matrix} -\rho_{d}\sigma_{x}\sigma_{wsm} & 0 \\ \rho\sigma_{x}\sigma_{bsm} & \rho_{d}\rho\sigma_{wsm} \sigma_{bsm} \end{matrix} & \begin{matrix} \sigma_{wsm}^{2} & -\rho_{d}\rho\sigma_{wsm}\sigma_{bsm} \\ -\rho_{d}\rho\sigma_{wsm}\sigma_{bsm} & \sigma_{bsm}^{2} \end{matrix} \end{matrix} \right)$ (A9)

This model requires that $\rho_{d}^{2}$ < 1/2. Violation of this condition leads to the covariance matrix losing the property of being positive definite, and would produce an expected mean square error in the ANCOVA that is < 0.

To translate the covariance matrix for these four variables into two observable dependent measures and the covariate, we note that the performance of subject *k* in within-subject Condition 1 ($y_{1,k})$ is the sum of the within-subject effect for that subject plus the effect of being subject *k*. Hence $y_{1,k}= {WLm}_{1,k}+{SLm}_{k}$, and the corresponding observable behavior for condition 2 is $y_{2,k}= {WLm}_{2,k}+{SLm}_{k}$.

The expected values of these three observable variables are: $\mu_{x}, \mu+W$, and $\mu$ - *W*, for $x_{k}, y_{1,k}$, and $y_{2,k}$, respectively. The covariance of the $y_{1,k}$ and the $x_{k}$ is

$Cov\left[ y_{1,k},x_{k} \right]=Cov\left[ {WLm}_{1,k},x_{k} \right]+Cov\left[ {SLm}_{k},x_{k} \right]= \rho_{d}\sigma_{x}\sigma_{wsm}+ \rho\sigma_{x}\sigma_{bsm}$ (A10)

By a similar argument,

$Cov\left[ y_{2,k},x_{k} \right]=Cov\left[ {WLm}_{2,k},x_{k} \right]+Cov\left[ {SLm}_{k},x_{k} \right]= -\rho_{d}\sigma_{x}\sigma_{wsm}+ \rho\sigma_{x}\sigma_{bsm}$ (A11)

To derive the covariance between $y_{1,k}$ and $y_{2,k}$ we note that

$Cov\left[ y_{1,k},y_{2,k} \right]=Cov\left[ \left( {WLm}_{1,k}+{SLm}_{k} \right), \left( {WLm}_{2,k}+{SLm}_{k} \right) \right]$

$= \left( \begin{aligned} Cov\left[ {WLm}_{1,k},{WLm}_{2,k} \right]+Cov\left[ {WLm}_{1,k},{SLm}_{k} \right] \\ +Cov\left[ {WLm}_{2,k},{SLm}_{k} \right]+Cov\left[ {SLm}_{k},{SLm}_{k} \right] \end{aligned} \right)$ $=\sigma_{bsm}^{2}$ (A12)

To complete the specification of the covariance matrix for the three observable variables we note

$$Var\left[ x_{k} \right]=\sigma_{x}^{2}$$

$$Var\left[ y_{1,k} \right]=Var\left[ {WLm}_{1,k} \right]+Var\left[ {SLm}_{k} \right]+2Cov\left[ {WLm}_{1,k},{SLm}_{k} \right]=\sigma_{bsm}^{2}+\sigma_{wsm}^{2}+2 \rho_{d}\rho\sigma_{wsm}\sigma_{bsm}$$

(A13)

$$Var\left[ y_{2,k} \right]=Var\left[ {WLm}_{2,k} \right]+Var\left[ {SLm}_{k} \right]+2Cov\left[ {WLm}_{2,k},{SLm}_{k} \right]=\sigma_{bsm}^{2}+\sigma_{wsm}^{2}-2 \rho_{d}\rho\sigma_{wsm}\sigma_{bsm}$$

Hence the covariance matrix for the three observable random variables is

$$\left( \begin{matrix} \sigma_{x}^{2} & \rho_{d}\sigma_{x}\sigma_{wsm}+ \rho\sigma_{x}\sigma_{bsm} & \rho\sigma_{x}\sigma_{bsm}-\rho_{d}\sigma_{x}\sigma_{wsm} \\ \rho_{d}\sigma_{x}\sigma_{wsm}+ \rho\sigma_{x}\sigma_{bsm} & \sigma_{bsm}^{2}+\sigma_{wsm}^{2}+2 \rho_{d}\rho\sigma_{wsm}\sigma_{bsm} & \sigma_{bsm}^{2} \\ \rho\sigma_{x}\sigma_{bsm}-\rho_{d}\sigma_{x}\sigma_{wsm} & \sigma_{bsm}^{2} & \sigma_{bsm}^{2}+\sigma_{wsm}^{2}-2 \rho_{d}\rho\sigma_{wsm}\sigma_{bsm} \end{matrix} \right) (A14)$$

The general linear model version^[[4]](#footnote-4)^ of the multi-normal model is

$y_{1,k}=\left( \alpha+\alpha_{d} \right)\left( x_{k}-\mu_{x} \right)+{SL}_{k}+{WL}_{1,k}$

$y_{2,k}=\left( \alpha-\alpha_{d} \right)\left( x_{k}-\mu_{x} \right)+{SL}_{k}+{WL}_{2,k}$ (A15)

The parameter, *α*, is the slope of the linear function relating the subject effect to the covariate in the population. The parameter *α_d_* is the amount by which the slope of the line relating the dependent variable to covariate is changed by Within Condition 1. In the general linear model the ${SL}_{k}, x_{k}, {WL}_{1,k}, \mathrm{and} {WL}_{2,k}$ are independent random normal variables with the following distributions

${WL}_{1,k}\sim N\left[ W,\sigma_{ws} \right], {WL}_{2,k}\sim N\left[ -W,\sigma_{ws} \right],{SL}_{k}\sim N\left[ \mu,\sigma_{bs} \right], x_{k}\sim N\left[ \mu_{x},\sigma_{x} \right]$ (A17)

It follows that

$y_{1,k}\sim N\left[ \mu+W,\sqrt{\left( \alpha+\alpha_{d} \right)^{2}\sigma_{x}^{2}+\sigma_{bs}^{2}+\sigma_{ws}^{2}} \right], y_{2,k}\sim N\left[ \mu-W,\sqrt{\left( \alpha-\alpha_{d} \right)^{2}\sigma_{x}^{2}+\sigma_{bs}^{2}+\sigma_{ws}^{2}} \right]$. (A18)

The covariance of $y_{1,k}$ and $x_{k}$ in the general linear model is

$\begin{matrix} Cov\left[y_{1,k}, x_{k}\right]=Cov\left[\left((\alpha+\alpha_{d}) \left(x_{k}-\mu_{x}\right)+SL_{k}+WL_{1,k}\right), x_{k}\right] \\ =Cov\left[\left((\alpha+\alpha_{d}) \left(x_{k}-\mu_{x}\right), x_{k}\right)\right]+Cov\left[SL_{k},x_{k}\right]+Cov\left[WL_{1,k},x_{k}\right] \\ =\left(\alpha+\alpha_{d}\right) \sigma_{x}^{2} \end{matrix}$ (A19)

A similar argument shows that the covariance of $y_{2,k}$ and $x_{k}$ in the linear regression model is

$Cov\left[y_{2,k}, x_{k}\right]=\left(\alpha-\alpha_{d}\right) \sigma_{x}^{2}$ (A20)

Finally the covariance of $y_{1,k}$ and $y_{2,k}$is

$Cov\left[ y_{1,k},y_{2,k} \right]=\left( \begin{aligned} Cov\left[ \left( \alpha+\alpha_{d} \right)\left( x_{k}-\mu_{x} \right),\left( \alpha-\alpha_{d} \right)\left( x_{k}-\mu_{x} \right) \right] \\ +Cov\left[ \left( \alpha+\alpha_{d} \right)\left( x_{k}-\mu_{x} \right),{SL}_{k} \right] \\ +Cov\left[ \left( \alpha+\alpha_{d} \right)\left( x_{k}-\mu_{x} \right),{WL}_{2,k} \right] \\ +Cov\left[ \left( \alpha-\alpha_{d} \right)\left( x_{k}-\mu_{x} \right),{SL}_{k} \right] \\ +Cov\left[ {SL}_{k},{SL}_{k} \right] \\ +Cov\left[ {SL}_{k},{WL}_{2,k} \right] \\ +Cov\left[ \left( \alpha-\alpha_{d} \right)\left( x_{k}-\mu_{x} \right),{WL}_{1,k} \right] \\ +Cov\left[ {SL}_{k},{WL}_{1,k} \right] \\ +Cov\left[ {WL}_{1,k},{WL}_{2,k} \right] \end{aligned} \right)=\left( \alpha^{2}-\alpha_{d}^{2} \right)\sigma_{x}^{2}+\sigma_{bs}^{2}$

(A21)

Hence the covariance matrix for the regression model is

$\left( \begin{matrix} \sigma_{x}^{2} & \left( \alpha+\alpha_{d} \right)\sigma_{x}^{2} & \left( \alpha-\alpha_{d} \right)\sigma_{x}^{2} \\ \left( \alpha+\alpha_{d} \right)\sigma_{x}^{2} & \sigma_{bs}^{2}+\sigma_{ws}^{2}+ \left( \alpha+\alpha_{d} \right)^{2}\sigma_{x}^{2} & \left( \alpha^{2}-\alpha_{d}^{2} \right)\sigma_{x}^{2}+\sigma_{bs}^{2} \\ \left( \alpha-\alpha_{d} \right)\sigma_{x}^{2} & \left( \alpha^{2}-\alpha_{d}^{2} \right)\sigma_{x}^{2}+\sigma_{bs}^{2} & \sigma_{bs}^{2}+\sigma_{ws}^{2}+ \left( \alpha-\alpha_{d} \right)^{2}\sigma_{x}^{2} \end{matrix} \right)$ (A22)

The relationships between the parameters of the general linear model to those of the multi-normal model are:

$\alpha=\frac{\rho\sigma_{bsm}}{\sigma_{x}}, \alpha_{d}=\frac{\rho_{d}\sigma_{wsm}}{\sigma_{x}}, \sigma_{ws1}=\sigma_{ws2}=\sigma_{wsm}\sqrt{1-2 \rho_{d}^{2}}$

(A23)

$\sigma_{bs}= \sqrt{\sigma_{bsm}^{2}\left( 1-\rho^{2} \right)+\rho_{d}^{2}\sigma_{wsm}^{2}}$

Given the relationships defined in A23, it is clear that covariance matrix for the general linear model of a within-subject Experiment with 2 levels (A22) is equivalent to the covariance matrix of the multi-normal model of such an experiment (A14). Hence the general linear model specified in A15 of a within-subject Experiment for a single group is equivalent to the multi-normal model of the same experiment.

**A4. A Mixed Design with 2 levels of a Between-Subjects factor and 2 levels of a Within-Subjects factor**

For a mixed model experiment with 2-Between × 2-Within factors, there is an extra parameter, namely the size of the interaction effect between the Within-subject and Between-subjects factors which is specified as *BW*. For level 1 of the between-subjects factor, there are two Within-subject levels. Let $y_{1,1,k} \mathrm{and}y_{1,2,k}$ specify the dependent score of the *k*th subject in Group 1, in Within-subject Conditions 1 and 2, respectively, where the first subscript identifies the group (Between-subjects level), and the second subscript the Within-subject level. The equivalent Within-subject Conditions for Group 2 are $y_{2,1,k} \mathrm{and}y_{2,2,k}$. In the multi-normal model of this experiment there are four latent Within-subject distributions (${WLm}_{1,1},{WLm}_{1,2}, {WLm}_{2,1}$, and ${WLm}_{2,2}$), where the first subscript specifies the level of the Between-subjects factor, and the second subscript the level of the Within-subject factor. The expected values of the four distributions are *W+BW*, -*W-BW*, *W –BW*, and *–W + BW* for ${WLm}_{1,1},{WLm}_{1,2}, {WLm}_{2,1}$, and ${WLm}_{2,2}$, respectively. Because there are two groups there are two levels of the latent variable for Subjects, ${SLm}_{1,k}$ and , ${SLm}_{2,k}$ for levels 1 and 2 of the Between-subjects factor, respectively, with means of *μ + B*, and *μ -B*. The standard deviations of the latent Within-subject and Between-subjects factors are assumed to be $\sigma_{wsm}$ and $\sigma_{bsm}$, respectively. The covariates for the two groups ($x_{1,k} \mathrm{and} x_{2,k}$) are assumed to be distributed as in Equation A8. Therefore the Within-subject covariance matrices for Groups 1 and 2 are both equal to the covariance matrix specified in Equation A9. Hence in the multi-normal model of this experiment,

$y_{1,1,k}={WLm}_{1,1,k}+{SLm}_{1,k}$

$y_{1,2,k}={WLm}_{1,2,k}+{SLm}_{1,k}$

(A24)

$y_{2,1,k}={WLm}_{2,1,k}+{SLm}_{2,k}$

$y_{2,2,k}={WLm}_{2,2,k}+{SLm}_{2,k}$

It can be shown that the multi-normal model of covariance defined in A24 corresponds to the general linear model specified in Equation 3 in the main body of the text, where Equation A23 specifies the relationships of $\alpha, \alpha_{d}, \sigma_{ws}$ and $\sigma_{bs}$ to $\rho, \rho_{d}, \sigma_{wsm}$ and $\sigma_{bsm}$.

**B. Expected values for an ANCOVA in three experimental designs involving covariance**

**B1. Expected values in a between-subjects design with 2 levels of the between-subjects factor**.

The first step in an ANCOVA is to center the dependent variable. The general linear model for a Between-subjects design with 2 levels, n measures per level, with the dependent variable, y, centered is

${yc}_{1,k}=B+\alpha\left( x_{1,k}-\frac{\bar{x_{1}}+\bar{x_{2}}}{2} \right)-\alpha\left( \frac{\mu_{x1}-\mu_{x2}}{2} \right)+e_{wg1,k}-\frac{\bar{e_{wg1}}}{2}-\frac{\bar{e_{wg2}}}{2}$

(B1)

${yc}_{2,k}=-B+\alpha\left( x_{2,k}-\frac{\bar{x_{1}}+\bar{x_{2}}}{2} \right)+\alpha\left( \frac{\mu_{x1}-\mu_{x2}}{2} \right)+e_{wg2,k}-\frac{\bar{e_{wg1}}}{2}-\frac{\bar{e_{wg2}}}{2}$

where *yc* is the centered dependent variable whose first subscript indicates the Between-subjects level with the second subscript indicating the subject in the group. *B* is the level 1 Between-subjects effect, *α* is the scale value relating the covariate to the dependent variable, *x_1,k_* is the covariate measure on the *k*th subject in group 1, and *x_2,k_* is the covariate measure on the *k*th subject in group 2. Both *x_1,k_* and *x_2,k_* are normally distributed random variables with means *μ_x1_* and *μ_x2_*, respectively, and a common standard deviation of *σ_x_*. The error terms *e_wg1,k_* and *e_wg2,k_* are also normally distributed with means equal to 0 and standard deviations equal to *σ_wg_*_._  To simplify the derivation of the expected values, we also define ${xc}_{1,k}=x_{1,k}-\bar{x_{1}}$, and ${xc}_{2,k}=x_{2,k}-\bar{x_{2}}$ so that

$\bar{{xc}^{2}}=\frac{\left( \sum_{k=1}^{n} {xc}_{1,k}^{2}+\sum_{k=1}^{n} {xc}_{2,k}^{2} \right)}{\left( 2 n \right)}, \bar{{yc}_{1}}=\left( \sum_{k=1}^{n} {yc}_{1,k} \right)/n$

$\bar{xc⦁yc}=\left( \sum_{k=1}^{n} {xc}_{1,k}{yc}_{1,k}+\sum_{k=1}^{n} {{xc}_{2,k}yc}_{2,k} \right)/\left( 2 n \right)$

and $\bar{xc⦁e}=\left( \sum_{k=1}^{n} {xc}_{1,k}e_{wg1,k}+\sum_{k=1}^{n} {{xc}_{2,k}e}_{wg2,k} \right)/\left( 2 n \right)$

We can then show that a standard ANCOVA analysis computes the sums of squares for this design as

${SS}_{Bet,ANCOVA}=\frac{n\left( \left( \bar{x_{1}}-\bar{x_{2}} \right) \bar{xc⦁yc}-2\bar{{yc}_{1}} \bar{{xc}^{2}} \right)^{2}}{2\bar{{xc}^{2}}\left( \bar{{xc}^{2}}+\frac{\left( \bar{x_{1}}-\bar{x_{2}} \right)^{2}}{4} \right)}, {SS}_{Bet,ANOVA}=2 n\left( \bar{{yc}_{1}} \right)^{2}$

(B2)

${SS}_{Cov}=\frac{2 n\left( \bar{xc⦁yc} \right)^{2}}{\bar{{xc}^{2}}}$

If we define ${SS}_{yc}=\sum_{k=1}^{n} {yc}_{1,k}^{2}+\sum_{k=1}^{n} {yc}_{2,k}^{2}$then

${SS}_{Error,ANOVA}={SS}_{yc}-{SS}_{Bet,ANOVA}, {SS}_{Error,ANCOVA}={SS}_{Error,ANOVA}-{SS}_{Cov}$ (B3)

We begin by defining the expected value of various terms with respect to *e*, or *x*, or both. When we are finding the expected value of a *quantity* with respect to *e*, we use the notation *E_e_*[*quantity*]. When we are finding the expected value of a quantity with respect to the covariate measure, *x*, we used the notation *E_x_*[*quantity*]. Finally, when we are defining the expected value with respect to both *e* and *x*, we use the notation *E*[*quantity*]. In doing so, it is advantageous to first note that

$E_{e}\left[ e_{wg1,k} \right]=E_{e}\left[ e_{wg2,k} \right]=E_{e}\left[ \bar{e_{wg1}} \right]=E_{e}\left[ \bar{e_{wg2}} \right]=0,$

$E_{e}\left[ {e_{wg1,k}}^{2} \right]= E_{e}\left[ {e_{wg2,k}}^{2} \right]=\sigma_{wg}^{2},E_{e}\left[ ({\bar{e_{wg1}})}^{2} \right]=E_{e}\left[ ({\bar{e_{wg2}})}^{2} \right]=\frac{\sigma_{wg}^{2}}{n},$

$E_{e}\left[ e_{wg1,k}\bar{e_{wg1}} \right]=E_{e}\left[ e_{wg2,k}\bar{e_{wg2}} \right]=\frac{\sigma_{wg}^{2}}{n},E_{e}\left[ \bar{xc⦁e} \right]=0,E_{e}\left[ \left( \bar{xc⦁e} \right)^{2} \right]=\frac{\sigma_{wg}^{2}\bar{{xc}^{2}}}{2 n}$

$E_{e}\left[ \bar{e_{wg1}}\left( \bar{xc⦁e} \right) \right]=E_{e}\left[ \bar{e_{wg2}}\left( \bar{xc⦁e} \right) \right]=0, E_{x}\left[ \bar{x_{1}} x_{1,k} \right]=\frac{\sigma_{x}^{2}}{n}+\mu_{x1}^{2},E_{x}\left[ \bar{x_{2}} x_{2,k} \right]=\frac{\sigma_{x}^{2}}{n}+\mu_{x2}^{2},$ (B4)

$E_{x}\left[ x_{1,k} \right]=E_{x}\left[ \bar{x_{1}} \right]=\mu_{x1},E_{x}\left[ x_{2,k} \right]=E_{x}\left[ \bar{x_{2}} \right]=\mu_{x2},E_{x}\left[ x_{1,k}^{2} \right]=\sigma_{x}^{2}+\mu_{x1}^{2},$

$E_{x}\left[ x_{2,k}^{2} \right]=\sigma_{x}^{2}+\mu_{x2}^{2},E_{x}\left[ \left( \bar{x_{1}} \right)^{2} \right]=\frac{\sigma_{x}^{2}}{n}+\mu_{x1}^{2},E_{x}\left[ \left( \bar{x_{2}} \right)^{2} \right]=\frac{\sigma_{x}^{2}}{n}+\mu_{x2}^{2},$

$E_{x}\left[ \bar{{xc}_{1}^{2}} \right]=E_{x}\left[ \bar{{xc}_{2}^{2}} \right]=E_{x}\left[ \bar{{xc}^{2}} \right]=\left( n-1 \right)\frac{\sigma_{x}^{2}}{n}$

First we note that sum of the squared centered dependent measures is

${SS}_{yc}=\sum_{k=1}^{n} {yc}_{1,k}^{2}+\sum_{k=1}^{n} {yc}_{2,k}^{2}$ (B5)

Since the *x_1,k_, x_2,k_, e_wg1_,* and *e_wg2_* are all independent random variables, we can expand ${SS}_{yc}$ and take the expected values of the terms in this expansion. We have done this using the Mathematica platform. A copy of this Mathematica program is available upon request from the corresponding author. The result is that

$EV\left[ {SS}_{yc} \right]=2 n B^{2}+\left( 2 n-1 \right)\alpha^{2}\sigma_{x}^{2}+\left( 2 n-1 \right)\sigma_{wg}^{2}$ (B6)

Next we expand ${(\bar{{yc}_{1}})}^{2}$ and determine the expected values of the individual terms in this expansion. This expansion yields

$E\left[ \left( \bar{{yc}_{1}} \right)^{2} \right]=B^{2}+\frac{1}{2 n}\alpha^{2}\sigma_{x}^{2}+\frac{1}{2 n}\sigma_{wg}^{2}$

(B7)

$E\left[ {SS}_{Bet,ANOVA} \right]=2 n E\left[ \left( \bar{{yc}_{1}} \right)^{2} \right]=2 n B^{2}+\alpha^{2}\sigma_{x}^{2}+\sigma_{wg}^{2}$

In determining the expected value for the covariate, we note that the denominator for the sum of squares for the covariate is devoid of any *e_wg_* terms. Hence, we first take the expected value of the numerator with respect to these error terms, i.e., we first expand ${(\bar{xc⦁yc})}^{2}$ and then determine its expected value with respect to the *e_wg_* terms. Next we divide it by the denominator for the sum of squares for the covariate, and then take its expected value with respect to the covariate terms to obtain the expected value of the sum of squares for the covariate.

$E_{e}\left[ \left( \bar{xc⦁yc} \right)^{2} \right]=\left( \bar{{xc}^{2}} \right)^{2}\alpha^{2}+\frac{\bar{{xc}^{2}} \sigma_{wg}^{2}}{2 n}$

$E_{e}\left[ {SS}_{Cov} \right]=2n \frac{E_{e}\left[ \left( \bar{xc⦁yc} \right)^{2} \right]}{\bar{{xc}^{2}}}=2 n \bar{{xc}^{2}} \alpha^{2}+\sigma_{wg}^{2}$ (B8)

$E\left[ {SS}_{Cov} \right]=2 \left( n-1 \right)\alpha^{2} \sigma_{x}^{2}+\sigma_{wg}^{2}$

Hence

$E\left[ {SS}_{Error,ANOVA} \right]=E\left[ {SS}_{yc} \right]-2 n E\left[ \left( \bar{{yc}_{1}} \right)^{2} \right]=2 \left( n-1 \right)\alpha^{2} \sigma_{x}^{2}+2 \left( n-1 \right) \sigma_{wg}^{2}$

(B9)

$E\left[ {SS}_{Error,ANCOVA} \right]= E\left[ {SS}_{Error,ANOVA} \right]- E\left[ {SS}_{Cov} \right]=\left( 2 n-3 \right)\sigma_{wg}^{2}$

Note that the denominator in the ANCOVA sum of squares for the Between-subjects Main Effect (see Equation B2) does not contain any within-group error terms, whereas the numerator does. Hence we first determine the expected value of the numerator with respect to these error terms, and then divide the numerator by the denominator of the sum of squares of the Between-subjects Main Effect. In determining the expected value of the numerator we first expand it, and then take the expected values of each of the terms with respect to within-group error. Therefore

$numerator=n\left( \left( \bar{x_{1}}-\bar{x_{2}} \right) \bar{xc⦁yc}-2\bar{{yc}_{1}} \bar{{xc}^{2}} \right)^{2}$ (B10)

and

$E_{e}\left[ numerator \right]=\frac{1}{2}\bar{{xc}^{2}}\left( 2 n \bar{{xc}^{2}} \left( 2 B-\alpha\left( \mu_{x1}-\mu_{x2} \right) \right)^{2}+\left( 4 \bar{{xc}^{2}}+\left( \bar{x_{1}}-\bar{x_{2}} \right)^{2} \right) \sigma_{wg}^{2} \right)$

$E_{e}\left[ {SS}_{Bet,ANCOVA} \right]$ $=\frac{\frac{1}{2}\bar{{xc}^{2}}\left( 2 n \bar{{xc}^{2}}\left( 2 B-\alpha\left( \mu_{x1}-\mu_{x2} \right) \right)^{2}+\left( 4 \bar{{xc}^{2}}+\left( \bar{x_{1}}-\bar{x_{2}} \right)^{2} \right) \sigma_{wg}^{2} \right)}{2 \bar{{xc}^{2}} \left( \bar{{xc}^{2}}+\frac{\left( \bar{x_{1}}-\bar{x_{2}} \right)^{2}}{4} \right)}$

(B11)

$E_{e}\left[ {SS}_{Bet,ANCOVA} \right]$ $=\frac{2 n \bar{{xc}^{2}}\left( 2 B-\alpha\left( \mu_{x1}-\mu_{x2} \right) \right)^{2}}{4 \bar{{xc}^{2}}+\left( \bar{x_{1}}-\bar{x_{2}} \right)^{2}}$ $+\sigma_{wg}^{2}$

$E_{e}\left[ {SS}_{Bet,ANCOVA} \right]$ $=\frac{n (n-1) \left( 2 B-\alpha\left( \mu_{x1}-\mu_{x2} \right) \right)^{2}}{2\left( n-1 \right)+ \frac{(n-1{)\left( \bar{x_{1}}-\bar{x_{2}} \right)}^{2}}{2 \bar{{xc}^{2}}}}$ $+\sigma_{wg}^{2}$

Recall that the square of a t-test of $\bar{x_{1}}- \bar{x_{2}}$ is defined as

$\frac{\left( \bar{x_{1}}-\bar{x_{2}} \right)^{2}}{\frac{1}{n(n-1)}\left( \sum\left( x_{1}-\bar{x_{1}} \right)^{2}+\sum\left( x_{2}-\bar{x_{2}} \right)^{2} \right)}=\frac{(n-1{)\left( \bar{x_{1}}-\bar{x_{2}} \right)}^{2}}{2 \bar{{xc}^{2}}}$ = *f* (B12)

Note that if $\mu_{d}=\mu_{x1}-\mu_{x2}\neq0$, then *f* has a non-central F-Ratio (NCF) Distribution with degrees of freedom equal to 1, and 2 (n-1), and a non-centrality parameter, $\lambda= \frac{n \mu_{d}^{2}}{2 \sigma_{x}^{2}}$. Hence, in general,

$E\left[ {SS}_{Bet,ANCOVA} \right]=\int_{f=0}^{\infty} \frac{n (n-1) \left( 2 B-\alpha\left( \mu_{x1}-\mu_{x2} \right) \right)^{2}}{2\left( n-1 \right)+ f}$ ${PDF}_{NCF}\left[ f \right]df+\sigma_{wg}^{2}$ (B13)

However, when $\mu_{d}=0$, *f* has a central F-Ratio (CF) distribution. In that case, the integral evaluates to

$E\left[ {SS}_{Bet,ANC}\left[ \mu_{d}=0 \right] \right]=\int_{f=0}^{\infty} \frac{4 n (n-1) B^{2}}{2\left( n-1 \right)+ f}{PDF}_{CF}\left[ f \right]df+\sigma_{wg}^{2}=\frac{4 n (n-1) B^{2}}{2 n-1}+\sigma_{wg}^{2}$(B14)

**B2. Expected Values in a single-group, Within-Subject Design with 2 levels.**

The derivations here assume that the covariate measures have been centered. The first step in a Within-participant design is to subtract the participant’s mean response from his or her responses. We use *yc* to indicate that the dependent variable, *y*, has been centered. When this is done, the *k*th measures in levels 1 and 2 of the Within-subject factor become

${yc}_{1,k}=W+ \alpha_{d}x_{k}-\alpha_{d}\mu_{x}+\frac{e_{ws1,k}}{2}-\frac{e_{ws2,k}}{2}$

(B15)

${yc}_{2,k}=-W- \alpha_{d}x_{k}+\alpha_{d}\mu_{x}-\frac{e_{ws1,k}}{2}+\frac{e_{ws2,k}}{2}$

Note that ${yc}_{2,k}= -{yc}_{1,k}$.

The within sums of squares for an ANOVA or an ANCOVA of data generated by this model are

${SS}_{Within,ANOVA}={SS}_{Within,ANCOVA}={SS}_{Within}=2 n \left( \bar{{yc}_{1}} \right)^{2}$

(B16)

${SS}_{W*Cov}=2 n \frac{\left( \bar{{yc}_{1}⦁xc} \right)^{2}}{\bar{{xc}^{2}}}$

where ${xc}_{k}= x_{k}-\bar{x}$is the centered covariate and

$\bar{{yc}_{1}}=\frac{\sum_{k=1}^{n} {yc}_{1,k}}{n}, \bar{{yc}_{1}⦁xc}=\frac{\sum_{k=1}^{n} {{xc}_{k}yc}_{1,k}}{n}, \bar{{xc}^{2}}=\frac{\sum_{k=1}^{n} {{xc}_{k}}^{2}}{n}$ .

If we define ${SS}_{yc}=\sum_{k=1}^{n} {yc}_{1,k}^{2}+\sum_{k=1}^{n} {yc}_{2,k}^{2}$then

${SS}_{Error,ANOVA}={SS}_{yc}-{SS}_{Within}, {SS}_{Error,ANCOVA}={SS}_{Error,ANOVA}-{SS}_{W*Cov}$ (B17)

Below are the expected values of various quantities, which are used in the derivation of the expected values of the sums of squares. Note that $\bar{e_{ws1}⦁xc}=(\sum_{k=1}^{n} e_{ws1,k} {xc}_{k})/n$, and that $\bar{e_{ws2}⦁xc}=(\sum_{k=1}^{n} e_{ws2,k} {xc}_{k})/2$.

$E_{e}\left[ e_{ws1,k} \right]=E_{e}\left[ e_{ws2,k} \right]=E_{e}\left[ \bar{e_{ws1}} \right]=E_{e}\left[ \bar{e_{ws2}} \right]=0,$

$E_{e}\left[ e_{ws1,k}^{2} \right]=E_{e}\left[ e_{ws2,k}^{2} \right]=\sigma_{ws}^{2}, E_{e}\left[ \left( \bar{e_{ws1}} \right)^{2} \right]=E_{e}\left[ \left( \bar{e_{ws2}} \right)^{2} \right]=\sigma_{ws}^{2}/n$

$E_{x}\left[ x_{k} \right]=E_{x}\left[ \bar{x} \right]=\mu_{x}, E_{x}\left[ x_{k}^{2} \right]=\sigma_{x}^{2}{+\mu_{x}^{2}, E}_{x}\left[ \left( \bar{x} \right)^{2} \right]=\frac{\sigma_{x}^{2}}{n}+\mu_{x}^{2},$ (B18)

$E_{x}\left[ \left( \bar{xc} \right)^{2} \right]=\left( n-1 \right)\frac{\sigma_{x}^{2}}{n}, E_{e}\left[ \bar{e_{ws1}⦁xc} \right]=E_{e}\left[ \bar{e_{ws2}⦁xc} \right]=0,$

$E_{e}\left[ \left( \bar{e_{ws1}⦁xc} \right)^{2} \right]=E_{e}\left[ \left( \bar{e_{ws2}⦁xc} \right)^{2} \right]=\frac{\sigma_{ws}^{2}\bar{{xc}^{2}}}{n}$

Since

${yc}_{1,k}^{2}={yc}_{2,k}^{2}, E\left[ {SS}_{yc} \right]=2\sum_{k=1}^{n} E\left[ {yc}_{1,k}^{2} \right]=2 n E\left[ {yc}_{1,k}^{2} \right]$ (B19)

we can simply expand ${yc}_{1,k}^{2}$ and determine the expected values of each of the terms with respect to the expected values of the within-subject error terms, and the covariates using the expected values in B18. When we do this we find that

$E\left[ {yc}_{1,k}^{2} \right]=W^{2}+\alpha_{d}^{2} \sigma_{x}^{2}+\frac{\sigma_{ws}^{2}}{2}, E\left[ {SS}_{yc} \right]={2 n W}^{2}+2 n \alpha_{d}^{2} \sigma_{x}^{2}+n\sigma_{ws}^{2}$ (B20)

To derive the expected sum of squares for the Within Main Effect (covariate centered) we expanded ${(\bar{{yc}_{1}})}^{2}$and used the expected values of the terms in B18 to show that

$E\left[ \left( \bar{{yc}_{1}} \right)^{2} \right]= W^{2}+\frac{\sigma_{ws}^{2}}{2 n}+\frac{\alpha_{d}^{2} \sigma_{x}^{2}}{n},$

(B21)

$E\left[ {SS}_{Within} \right]=2 nE\left[ \left( \bar{{yc}_{1}} \right)^{2} \right]=2 n W^{2}+2 \alpha_{d}^{2} \sigma_{x}^{2}+\sigma_{ws}^{2}$

To find the expected sum of squares for the Within*Covariate interaction we note that

$E_{e}\left[ {SS}_{W*C} \right]=2 n \frac{E_{e}\left[ \left( \bar{xc⦁{yc}_{1}} \right)^{2} \right]}{\bar{{xc}^{2}}}=\frac{2 n \alpha_{d}^{2}\left( \bar{{xc}^{2}} \right)^{2}+\bar{{xc}^{2}} \sigma_{ws}^{2}}{\bar{{xc}^{2}}}=2 n \alpha_{d}^{2} \bar{{xc}^{2}}+\sigma_{ws}^{2}$

(B22)

$E\left[ {SS}_{W*C} \right]=E_{x}\left[ 2 n \alpha_{d}^{2} \bar{{xc}^{2}}+\sigma_{ws}^{2} \right]=2 \left( n-1 \right)\alpha_{d}^{2}\sigma_{x}^{2}+\sigma_{ws}^{2}$

It follows that if no covariate is included in the model, the expected value of the error sum of squares without the covariate (${SS}_{Error, ANOVA}$) becomes

$E\left[ {SS}_{Error,ANOVA} \right]=E\left[ {SS}_{yc} \right]-E\left[ {SS}_{Within} \right]=2 \left( n-1 \right)\alpha_{d}^{2}\sigma_{x}^{2}+\left( n-1 \right)\sigma_{ws}^{2}$ (B23)

Hence, the expected value of the Error Sum of Squares in the ANCOVA is

$\left[ {SS}_{Error,ANCOVA} \right]=E\left[ {SS}_{Error,ANOVA} \right]-E\left[ {SS}_{W*C} \right]= \left( n-2 \right)\sigma_{ws}^{2}$ (B24)

**B3. The Expected Value of the Between*Within Interaction in a 2-Between X 2-Within Design**

When the measures in this design have been centered within each subject, the model for this design becomes

${yc}_{1,1,k}=W+BW+ \alpha_{d}\left( x_{1,k}-\mu_{x1} \right)+\frac{e_{ws1,1,k}}{2}-\frac{e_{ws1,2,k}}{2}$

${yc}_{1,2,k}=-W-BW- \alpha_{d}\left( x_{1,k}-\mu_{x1} \right)-\frac{e_{ws1,1,k}}{2}+\frac{e_{ws1,2,k}}{2}$

(B25)

${yc}_{2,1,k}=W-BW+ \alpha_{d}\left( x_{2,k}-\mu_{x2} \right)+\frac{e_{ws2,1,k}}{2}-\frac{e_{ws2,2,k}}{2}$

${yc}_{2,2,k}=-W+BW- \alpha_{d}\left( x_{2,k}-\mu_{x2} \right)-\frac{e_{ws2,1,k}}{2}+\frac{e_{ws2,2,k}}{2}$

where $e_{ws}$ is within-subject error, the first subscript of *yc* specifies the group, and the second subscript, the within-subject condition. We define

${xc}_{1,k}=x_{1,k}-\bar{x_{1}}, {xc}_{2,k}=x_{2,k}-\bar{x_{2}},\bar{{xc}^{2}}=\frac{\sum_{k=1}^{n} {xc}_{1,k}^{2}+\sum_{k=1}^{n} {xc}_{1,k}^{2}}{2 n}, \bar{{yc}_{W1}⦁xc}=\frac{\sum_{i=1}^{2} \sum_{k=1}^{n} {xc}_{i,k} {yc}_{i,1,k}}{2 n}$

Given these definitions, the various sums of squares computed in the within portion of an ANOVA and ANCOVA are:

${SS}_{Within,ANOVA}={SS}_{Within,ANCOVA}= n \left( \bar{{yc}_{1,1}}+\bar{{yc}_{2,1}} \right)^{2}$

${SS}_{B*W,ANOVA}= n \left( \bar{{yc}_{1,1}}-\bar{{yc}_{2,1}} \right)^{2}$

${SS}_{W*C}=\frac{4 n \left( \bar{{yc}_{W1}⦁xc} \right)^{2}}{\bar{{xc}^{2}}}$ , ${SS}_{yc}=\sum_{k=1}^{n} \left( {yc}_{1,1,k}^{2}+{yc}_{1,2,k}^{2}+{yc}_{2,1,k}^{2}+{yc}_{2,2,k}^{2} \right)$

${SS}_{B*W,ANCOVA}=\frac{4 n \left( -\bar{{yc}_{1,1}}\bar{{xc}^{2}}+\bar{{yc}_{2,1}}\bar{{xc}^{2}}+\left( \bar{x_{1}}-\bar{x_{2}} \right) \bar{{yc}_{W1}⦁xc} \right)^{2}}{\left( \left( \bar{x_{1}}-\bar{x_{2}} \right)^{2}+4 \bar{{xc}^{2}} \right) \bar{{xc}^{2}}}$ (B26)

${SS}_{Error,ANOVA}={SS}_{yc}-{SS}_{Within,ANOVA}-{SS}_{B*W,ANOVA}$

${SS}_{Error,ANCOVA}={SS}_{Error,ANOVA}-{SS}_{W*Cov}$

As we did in B1 and B2, we expand each of the formulas for computing the sums of squares and then determine the expected values of each of the terms. Note that the formulas for the sum of squares for the W*Cov and B*W interactions contain both a numerator and denominator. In both cases, the denominator does not contain any within-subject error terms. Hence we first take the expected values of the terms in the numerator. In the case of the W*Cov interaction, we follow this up by taking the expected value with respect to the covariate, *x*. In the case of the B*W interaction sum of squares, the expected value of the numerator with respect to within-subject error is

$E_{e}\left[ numer \right]=\bar{{xc}^{2}}\left( 4 n \bar{{xc}^{2}} \left( 2 BW-\alpha_{d}\left( \mu_{x1}-\mu_{x2} \right) \right)^{2}+\left( 4 \bar{{xc}^{2}}+\left( \bar{x_{1}}-\bar{x_{2}} \right)^{2} \right) \sigma_{ws}^{2} \right)$ (B27)

Dividing this expected value by the denominator, rearranging terms, and applying B12 yields

$E_{e}\left[ {SS}_{B*W,ANCOVA} \right]$ $=\frac{2 n (n-1) \left( 2 BW-\alpha_{d}\left( \mu_{x1}-\mu_{x2} \right) \right)^{2}}{2\left( n-1 \right)+f}$ $+\sigma_{ws}^{2}$ (B28)

Note that unless $\mu_{d}=\mu_{x1}-\mu_{x2}=0$, then *f* has a non-central F-Ratio (NCF) Distribution with degrees of freedom equal to 1, and 2 (n-1), and a non-centrality parameter, $\lambda= \frac{n \mu_{d}^{2}}{2 \sigma_{x}^{2}}$. Hence, in general,

$E\left[ {SS}_{B*W,ANCOVA} \right]$ $=\int_{f=0}^{\infty} \frac{2 n (n-1) \left( 2 BW-\alpha_{d}\left( \mu_{x1}-\mu_{x2} \right) \right)^{2}}{2\left( n-1 \right)+ f}$ ${PDF}_{NCF}\left[ f \right]df+\sigma_{ws}^{2}$ (B29)

depends on the value of $\mu_{d}$. However, when $\mu_{d}$ = 0, *f* has a central F-Ratio Distribution. In that case, the integral evaluates to

$E\left[ {SS}_{B*W,ANCOVA}\left[ \mu_{d}=0 \right] \right]=\int_{f=0}^{\infty} \frac{8 n \left( n-1 \right){BW}^{2}}{2\left( n-1 \right)+ f}{PDF}_{CF}\left[ f \right]df+\sigma_{ws}^{2}$ (B30)

$=\frac{8 n (n-1) {BW}^{2}}{2 n-1}+\sigma_{ws}^{2}$

All of these calculations were checked using Mathematica, and verified using Monte Carlo techniques. These programs are available upon request from the corresponding author.

1. The reason why the between-subject variances differ between the bi-normal and the general linear model is that the subject effect is correlated with the covariate in the bi-normal model, whereas the effect of the covariate in the general linear model is expressed independently of the between-subject variance as shown in A4. In the general linear model, the subject effect is represented by ${SL}_{k}$. [↑](#footnote-ref-1)
2. The general linear model can also be written as $y_{k}=\mu+\alpha\left( x_{k}-\mu_{x} \right)+e_{k}$, where the error term, $e_{k}$, is normally distributed with a mean of 0 and a standard deviation equal to $\sqrt{\sigma_{ws}^{2}+\sigma_{bs}^{2}}$. [↑](#footnote-ref-2)
3. The general linear model can also be written as

   $y_{1,k}=\mu+B+\alpha\left( x_{1,k}-\mu_{x1} \right)+e_{1,k}$

   $y_{2,k}=\mu-B+\alpha\left( x_{2,k}-\mu_{x2} \right)+e_{2,k}$

   , where both $e_{1,k} \mathrm{and} e_{2,k} have means of 0, and standard deviations= \sqrt{\sigma_{ws}^{2}+\sigma_{bs}^{2}}.$ It is this latter form, rather than the form expressed in A9 which appears in the main text as Equation 1. [↑](#footnote-ref-3)
4. The general model can also be written as

   $y_{1,k}=\mu+W+\left( \alpha+\alpha_{d} \right)\left( x_{k}-\mu_{x} \right)+S_{k}+e_{ws1,k}$

   $y_{2,k}=\mu-W+\left( \alpha-\alpha_{d} \right)\left( x_{k}-\mu_{x} \right)+S_{k}+e_{ws2,k}$

   where both $e_{ws1,k} \mathrm{and} e_{ws2,k} have means of 0, and standard deviations=\sigma_{ws}$ and $S_{k}$ has a mean of 0 and a standard deviation of $\sigma_{bs}$. It is this latter form, rather than the form expressed in A15 which appears in the main text as Equation 2.

   [↑](#footnote-ref-4)
